# Epigenetic Targeting of Senescent Cells Prevents the Deleterious Effects of Obstructive Sleep Apnea on Growing Skeleton

**DOI:** 10.1002/advs.202502697

**Published:** 2025-12-12

**Authors:** Xiaonan Liu, Peilin Zhang, Zhongyi Su, Yong Feng, Zhenger Zhou, Sa Pang, Yicheng Wang, Jiacheng Hu

**Affiliations:** ^1^ Department of Orthopaedics Shanghai Sixth People's Hospital Affiliated to Shanghai Jiao Tong University School of Medicine Shanghai 200233 P. R. China; ^2^ Department of Orthopaedic Surgery Chongqing Emergency Medical Center Chongqing University Central Hospital Chongqing 400014 P. R. China; ^3^ Department of Otolaryngology‐Head and Neck Surgery Shanghai Sixth People's Hospital Affiliated to Shanghai Jiao Tong University School of Medicine Shanghai 200233 P. R. China

**Keywords:** bone growth, chronic intermittent hypoxia, H3K27me3, HIF‐1α, obstructive sleep apnea, osteoprogenitor senescence

## Abstract

Obstructive Sleep Apnea Syndrome (OSAS) is a common sleep disorder characterized by chronic intermittent hypoxia (CIH), which has been increasingly recognized for its systemic effects on pediatric skeletal development. However, the mechanism by which CIH influences bone growth and homeostasis remains largely unexplored. In this study, it is demonstrated that CIH exposure in young murine models induces cellular senescence within the metaphysis of long bones, resulting in compromised bone formation and growth retardation. Through single cell sequencing and in situ immunostaining, it is identified that the senescent cells predominantly consist of osteoprogenitors. Mechanistically, CIH enhances the activity of hypoxia‐inducible factor 1‐alpha (HIF‐1α) in osteoprogenitors and subsequently downregulates trimethylation of histone H3 at lysine 27 (H3k27me3) through the suppression of polycomb histone methyltransferase enhancer of zeste homolog 2 (EZH2), thereby facilitating the expression of senescence‐associated genes. Employing both genetic and pharmacological strategies, it is demonstrated that the restoration of H3K27me3 levels via UTX inhibition (achieved through in vivo knockout or GSK‐J4 treatment) effectively prevents CIH‐induced senescence, promotes osteogenesis, and alleviates bone loss and growth retardation. These findings elucidate a novel epigenetic mechanism that underlies the skeletal impairments associated with CIH and underscore the therapeutic potential of targeting histone methylation to mitigate hypoxia‐induced bone defects.

## Graphical abstract

Chronic intermittent hypoxia induces premature senescence of osteoprogenitor cells in long bone metaphysis via HIF1α‐mediated EZH2‐H3k27me3 downregulation, leading to impaired bone growth. Restoration of H3K27me3 level via UTX inhibition rescues osteogenesis and growth impairment in young mice.

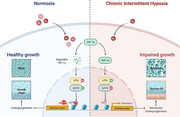

## 1 Introduction

Obstructive Sleep Apnea Syndrome (OSAS) is a prevalent and chronic sleep‐related breathing disorder characterized by recurrent episodes of upper airway obstruction during sleep, leading to chronic intermittent hypoxia (CIH) and subsequent physiological disturbances.^[^ ^1^, ^2^, ^3^, ^4^, ^5^ ^]^ It is estimated that ≈2–4% of children are affected by OSAS, with many remaining untreated for prolonged periods.^[^ ^6^ ^]^ Emerging evidence suggests that long‐term CIH exposure has significant systemic effects on children, impacting cardiovascular health, metabolic function, and neurocognitive performance.^[^ ^7^, ^8^, ^9^, ^10^ ^]^ While the impact of CIH on these systems is well‐documented, its effects on bone development in children have received less attention.^[^ ^11^, ^12^ ^]^ During critical periods of skeletal growth, such as childhood, CIH can impair bone mass acquisition, leading to growth retardation and an increased risk of fractures later in life.^[^ ^13^, ^14^, ^15^, ^16^, ^17^ ^]^ Despite these concerns, research on how OSAS affects bone growth and homeostasis remains limited, warranting further investigation into its potential long‐term skeletal consequences.

Bone growth and maintenance depend on the highly coordinated activities of osteoblasts, osteoclasts, and osteocytes, all of which are meticulously regulated by both systemic and local factors.^[^ ^18^ ^]^ The process of endochondral ossification, responsible for longitudinal bone growth, is particularly sensitive to disruptions in oxygen homeostasis.^[^ ^19^ ^]^ Both chronic and intermittent hypoxia have been shown to affect osteoblast differentiation, chondrocyte proliferation, and angiogenic signaling, ultimately compromising skeletal development.^[^ ^20^, ^21^ ^]^ While previous research has highlighted the detrimental effects of hypoxia on bone cells, the specific implications of CIH for skeletal growth, particularly during critical developmental periods, remain inadequately understood.

Hypoxia‐inducible factor‐1 alpha (HIF‐1α) is a principal regulator of the cellular response to hypoxia, mediating adaptive responses to low oxygen levels.^[^ ^22^ ^]^ Under hypoxic conditions, HIF‐1α stabilizes and activates target genes involved in angiogenesis, metabolism, and cell survival.^[^ ^23^, ^24^, ^25^ ^]^ In the context of bone biology, HIF‐1α plays a dual role: it promotes vascularization and osteogenesis under physiological conditions; however, prolonged or excessive activation can disrupt the balance between bone formation and resorption.^[^ ^26^ ^]^ Given that CIH results in repeated cycles of hypoxia and reoxygenation, it is plausible that this pattern of oxygen fluctuation may uniquely influence HIF‐1α activity, thereby affecting bone remodeling in a distinct manner.

Cellular senescence is characterized by irreversible cell cycle arrest induced by various stressors, including oxidative stress, DNA damage, and hypoxia.^[^ ^27^ ^]^ Senescent cells are marked by the expression of specific senescence‐associated markers, such as p16INK4a, p21Cip1, and senescence‐associated β‐galactosidase (SA‐βgal).^[^ ^28^ ^]^ Although cellular senescence is classically associated with aging, recent evidence has highlighted that premature senescence can be triggered by environmental stressors, including oxidative stress, DNA damage, and hypoxia, even during early life stages. This concept has been extended to developmental disorders and skeletal growth failure, where stress‐induced senescence impairs the function of progenitor cells and compromises tissue regeneration. Importantly, a recent study by Badran et al. demonstrated that clearance of senescent cells using Navitoclax (NAV) led to a reversal of end‐organ dysfunction in the OSAS‐CIH model, underscoring the pathological role of CIH‐induced senescence even in non‐aged models.^[^ ^29^ ^]^ Furthermore, senescent cells are known to secrete a variety of pro‐inflammatory cytokines and matrix‐degrading enzymes, collectively referred to as the senescence‐associated secretory phenotype (SASP), which may exacerbate bone loss and impede bone repair mechanisms.^[^ ^30^ ^]^ Mechanism‐wise, epigenetic regulation is likely to play a significant role in the induction of the senescence phenotype.^[^ ^31^, ^32^ ^]^ Epigenetic modifications, including DNA methylation and histone modifications, are essential regulators of gene expression and cellular function.^[^ ^33^, ^34^ ^]^ Among the most extensively studied histone modifications is the trimethylation of histone H3 at lysine 27 (H3K27me3), a process catalyzed by the polycomb repressive complex 2 (PRC2) and its core component, enhancer of zeste homolog 2 (EZH2).^[^ ^35^, ^36^ ^]^ H3K27me3 is typically associated with gene repression and has been shown to be critical for maintaining cellular proliferation and preventing aberrant cell states in various diseases.^[^ ^37^, ^38^ ^]^ The loss of H3K27me3 has been correlated with cellular senescence and the dysregulation of key signaling pathways integral to bone metabolism.^[^ ^39^, ^40^ ^]^

In this study, we utilized a murine model of CIH to investigate its effects on bone growth and homeostasis. Through the application of Micro‐CT, histological analyses, immunofluorescence staining, and single‐cell RNA sequencing, we demonstrated that CIH induces senescence in osteoprogenitors within long bones, resulting in reduced osteogenic activities in the spongiosa region. The activation of HIF‐1α under CIH conditions was found to decrease the H3K27me3 mark, leading to cellular senescence of osteoprogenitors. Furthermore, the inhibition of osteoprogenitor senescence through the manipulation of epigenetic factors was shown to rescue bone growth and mitigate bone loss impaired by CIH.

## 2 Results

### 2.1 CIH Induces Bone Loss and Growth Retardation in Young Mice

**Figure 1:**
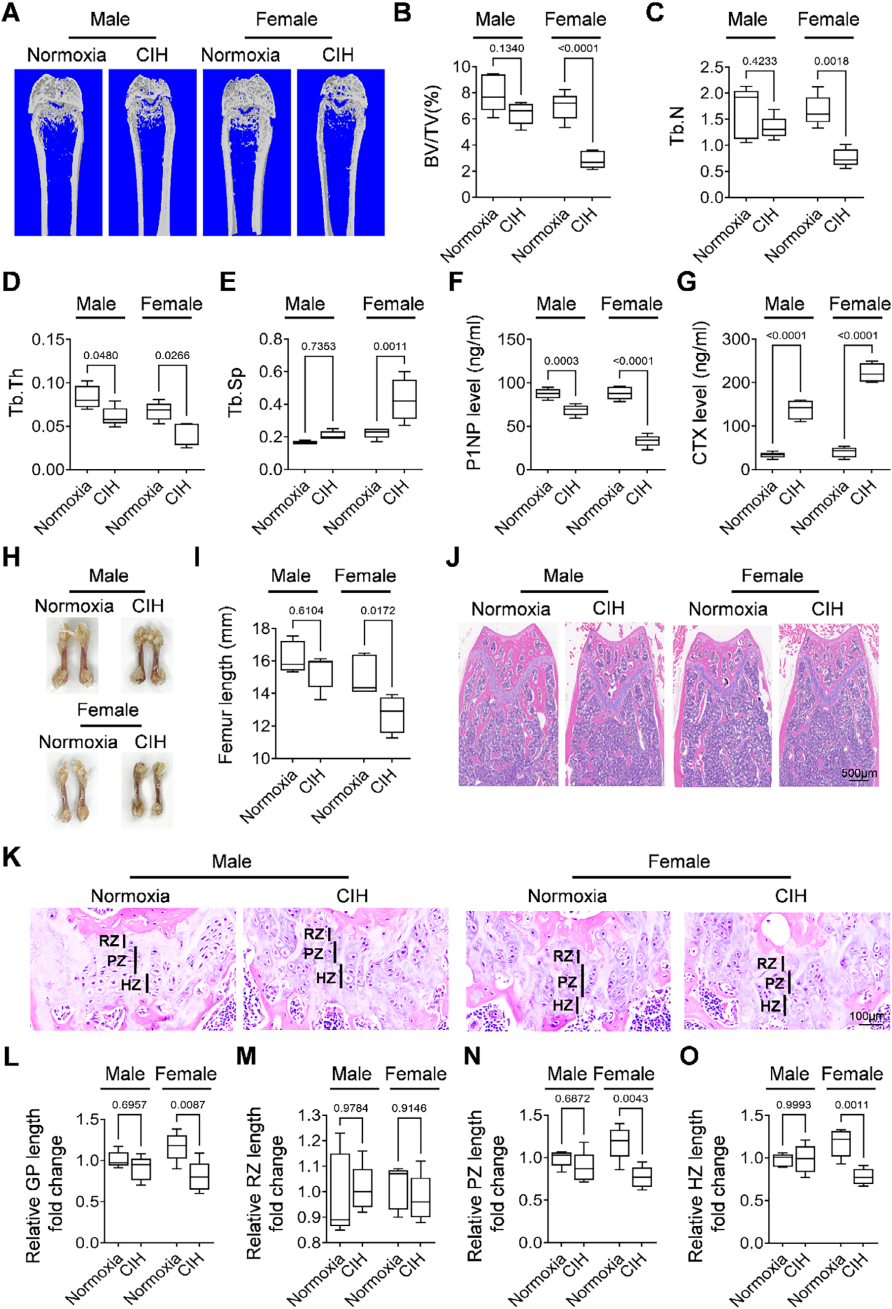
CIH induces bone loss and growth retardation in young mice. Three‐week‐old male or female wild‐type (WT) mice were subjected to CIH or normoxia condition for four weeks. A–E) Representative micro‐CT images of both male and female mice were shown in (A) with quantitative analysis of BV/TV (B), Tb. N (C), Tb. Th (D) and Tb. Sp (E). F,G) The serum concentrations of P1NP and CTX of both male and female mice were measured by ELISA. General view of the femurs was shown in H), and the length of the femurs were measured in I). J,K) H&E staining was performed to show the general morphology of the femoral bone and growth plate. RZ, resting zone; PZ, proliferating zone; HZ, hypertrophic zone; GP, growth plate. The fold change of GP length, RZ length, PZ length, and HZ length was quantified in L–O). *n* = 5‐6 mice Data were represented as mean ± SD. *p* values were determined by one‐way ANOVA.

To investigate the effects of CIH on young mice's bone growth, we subjected three‐week‐old mice to either CIH or normoxia conditions for a duration of four weeks. Given that male and female bones exhibit distinct developmental trajectories, we assessed the bone phenotype in mice of both sexes. Micro‐computed tomography (Micro‐CT) analysis revealed that CIH treatment led to a decrease in femoral bone mass in both sexes, as illustrated in **Figure** **1**A. Specifically, analysis of trabecular bone volume (BV/TV) indicated that the four‐week CIH treatment decreased femoral bone mass in female mice by 58.77% ± 9.59, whereas male mice experienced a reduction of 19.21% ± 10.70 (Figure 1B). Further examination demonstrated that CIH treatment resulted in a decrease in trabecular bone number (Tb. N) (Figure 1C), a decrease in trabecular bone thickness (Tb. Th) (Figure 1D), and an increase in trabecular separation (Tb. Sp) (Figure 1E) in the femoral trabecular bone of female mice. Conversely, in male mice, Tb. N and Tb. Sp remained unchanged (Figure 1C,E), while a decrease in trabecular bone thickness (Tb. Th) (Figure 1D) was observed. These findings suggested that CIH induced a bone loss phenotype in young mice in both sexes and affected bone growth in male and female mice in distinct manners. ELISA measurement of bone turnover markers N‐terminal propeptide of type I procollagen (P1NP) and C‐terminal telopeptide of type I collagen (CTX) suggested that CIH treatment inhibited bone formation while promoted bone resorption in both sexes (Figure 1F,G). Gross and histological evaluations using hematoxylin and eosin (H&E) staining of femoral bone sections corroborated our previous findings, revealing significant reductions in both femoral length and bone mass in female mice (Figure 1H–J). We further examined the length of the growth plate following CIH treatment in both male and female mice. H&E staining indicated a shorter length of the distal femoral growth plate in female mice subjected to CIH treatment (Figure 1K). Importantly, CIH treatment resulted in a decrease in growth plate length by 31.34% ± 15.85 in female mice, while the length of the growth plate reduced by 10.63% ± 8.74 in male mice (Figure 1L). The length of the resting zone remained unaffected in both sexes (Figure 1M). The lengths of the proliferative and hypertrophic zones were slightly reduced without a statistical difference in male mice treated with CIH, while female mice exhibited decreases of 34.41% ± 10.76 and 24.83% ± 11.96, respectively (Figure 1N,O). To investigate whether systemic hormonal changes may contribute to the observed sex differences in CIH‐induced bone loss in young mice, we measured serum estradiol levels in female mice after 4 weeks of CIH exposure. Compared to normoxia controls, CIH‐treated female mice exhibited a modest but statistically significant reduction in circulating estradiol levels (Figure S1A, Supporting Information). This result suggested that CIH may perturb estrogen homeostasis, which could contribute to the more severe bone phenotype observed in female mice. Collectively, these data suggest that CIH significantly impacts longitudinal bone growth and bone formation in both male and female young mice, with a more pronounced effect observed in female mice.

### 2.2 CIH Induces Osteoprogenitor Senescence in Growing Long Bone

**Figure 2:**
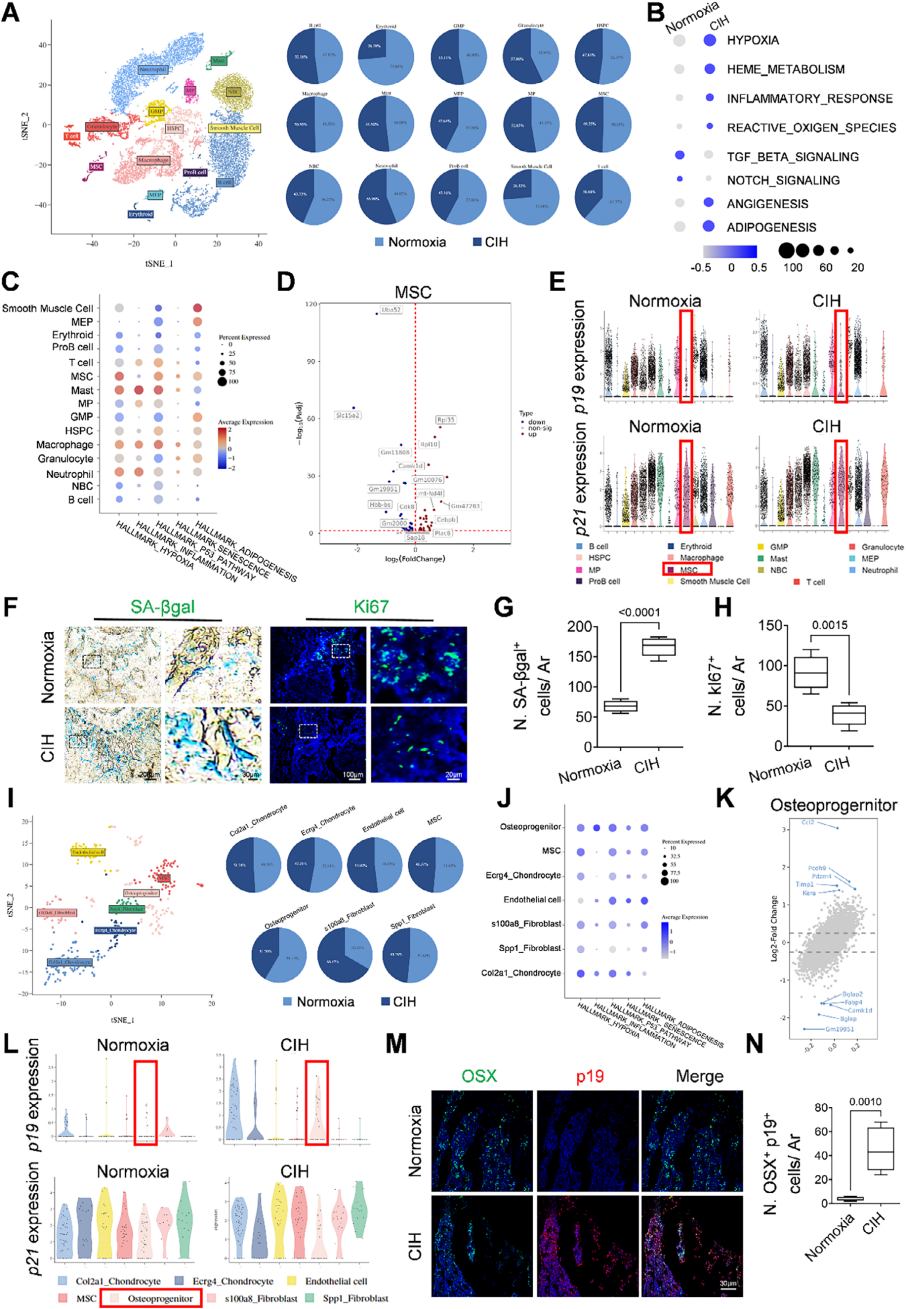
CIH induces osteoprogenitor senescence in growing long bone. A) Bioinformatics analysis of scRNA‐seq of bone marrow cells isolated from three‐week‐old female WT mice subjected to CIH or normoxia condition for four weeks. The percentage of different populations were shown in the right panel. B) Pathway enrichment analysis between Normoxia group VS CIH group. C) Bubble plot showing enrichment expressions of the selected hallmark gene sets in each population. D) Volcano plot showing the differentially expressed genes in the MSC cluster between Normoxia group VS CIH group in female mice. E) Violin plots of gene expression of *p19* and *p21* in different cell populations between the Normoxia group VS CIH group in female mice. Three‐week‐old female WT mice were subjected to CIH or normoxia condition for four weeks. F) Representative images of SA‐βGal staining and immunofluorescence staining of Ki67 in the distal femur section with quantification of the number of SA‐βGal^+^ cells per mm^2^ tissue area (N. SA‐βGal^+^ cells/ Ar) in G) and quantification of the number of Ki67^+^ cells per mm^2^ tissue area (N. Ki67^+^ cells/ Ar) in H). I) Bioinformatics analysis of scRNA‐seq of MSC subclusters. J) Bubble plot showing enrichment expressions of the selected hallmark gene sets in MSC subclusters. K) Scatter plot displaying the differentially expressed genes in the Osteoprogenitor subcluster compared between Normoxia group VS CIH group. L) Violin plots of gene expression of *p19* and *p21* in MSC subclusters between Normoxia group VS CIH group. M.N) Representative co‐immunofluorescence staining of OSX and p19 in female distal femur section (M) and analysis of cell number per mm^2^ tissue area (N. OSX^+^ p19^+^ cells/ Ar) (N). *n* = 5–6 mice. Data were represented as mean ± SD. *p* values were determined by unpaired *t*‐tests.

To deepen our understanding of the mechanisms associated with CIH‐induced mineral acquisition and bone growth impairment, we performed single‐cell RNA‐sequencing analysis on the isolated cells from the spongiosa region of the femoral bone in young female mice, comparing a 4‐week normoxia group with a 4‐week CIH group. Unbiased clustering of the cells, as determined by t‐Distributed Stochastic Neighbor Embedding (t‐SNE) analyses, revealed fifteen distinct clusters (**Figure** **2**A). A slight decrease in the fraction of the mesenchymal stem cell (MSC) cluster was noted, declining from 1.93% to 1.79% of all cells in the CIH group relative to the normoxia group (Figure 2A). Signaling pathway analyses indicated that CIH treatment across all populations was linked to increased hypoxia and inflammatory response (Figure 2B). Notably, CIH treatment led to the negative regulation of TGF‐β and Notch signaling pathways, which were linked to the activities of increased angiogenesis and adipogenesis (Figure 2B). We further conducted signaling pathway enrichment analyses on the fifteen identified clusters (Figure 2C). Importantly, CIH treatment significantly elevated the expression levels of hypoxia‐related genes within the MSC cluster. CIH treatment also activated genes associated with the p53 pathway, senescence, and adipogenesis activity in the MSC cluster, suggesting a substantial impact of CIH on the proliferation and differentiation of MSCs. Differential gene expression (DEG) analysis revealed an overexpression of the adipogenesis marker *Cebpb* and a significant reduction in the expression of the proliferative marker *Cdk8* within the MSC cluster (Figure 2D). We investigated the expression of senescence‐related genes, observing elevated levels of *p19* and *p21* in the MSC cluster of the CIH group compared to the normoxia group, suggesting increased senescence phenotype in this cluster (Figure 2E). To validate the CIH‐induced senescence phenotype, we performed senescence‐associated β‐galactosidase (SA‐βgal) staining on femoral bone sections. While a limited number of SA‐βgal‐positive cells were present in the normoxia group, CIH treatment significantly increased the number of SA‐βgal‐positive cells within the spongiosa area (Figure 2F,G). Immunofluorescence staining revealed a decrease in Ki67 levels (Figure 2F,H) in the CIH group compared to the normoxia group, indicating the onset of a senescence cell cycle arrest phenotype. To elucidate which cell type undergoes senescence during CIH treatment, we further identified the subcluster populations within the MSC cluster. A total of seven clusters were identified, including two chondrocyte subclusters (Col2a1^+^, Ecrg4^+^), an endothelial cell subcluster, a MSC subcluster, an osteoprogenitor subcluster, and two fibroblast subclusters (S100a8^+^, Spp1^+^) (Figure 2I). Pathway enrichment analyses indicated that CIH treatment upregulates hypoxia‐related genes in osteoprogenitors (Figure 2J). Furthermore, CIH treatment also upregulated the p53 pathway, senescence pathway, and adipogenesis pathway in osteoprogenitors (Figure 2J). Notably, the expression level of a classic SASP component *Ccl2* was elevated while the level of osteogenesis‐related genes: *Bglap*, *Camk1d* were decreased in comparison between the normoxia and CIH groups (Figure 2K). Osteoprogenitors exhibited significantly higher expression levels of *p19* and a mild increase of *p21* expression revealed by single‐cell RNA sequencing data (Figure 2L). At last, we confirmed the expression of p19^+^ osteoprogenitors through immunofluorescence staining of femoral bone sections (Figure 2M,N). As anticipated, p19 expression was minimally detected in the normoxia group; however, CIH treatment significantly increased p19 levels in OSX^+^ osteoprogenitors. Importantly, we also found that senescence markers such as SA‐β‐gal were also elevated in osteoprogenitor cells from male mice, indicating that premature senescence is a shared consequence of CIH across genders (Figure S2A,B, Supporting Information). Thus, these findings substantiate that osteoprogenitors undergo senescence under CIH conditions.

### 2.3 HIF1α Activation Induces Osteoprogenitor Senescence via Loss of EZH2‐H3K27me3

**Figure 3:**
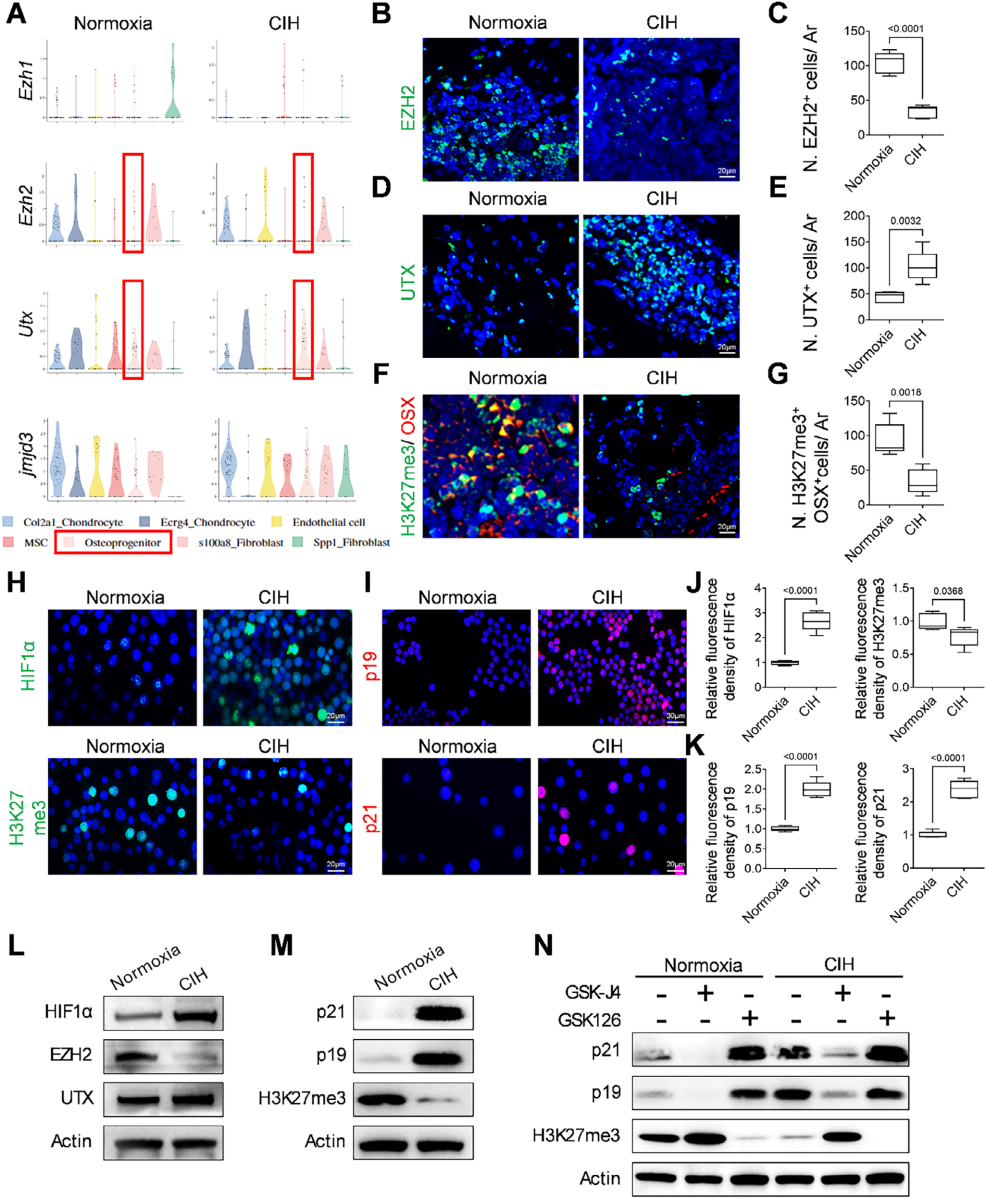
HIF1α activation induces osteoprogenitor senescence via loss of Ezh2‐H3K27me3. A) Violin plots of gene expression of *Ezh1, Ezh2, Utx*, and *Jmjd3* in MSC subclusters between the Normoxia group VS CIH group. Three‐week‐old female WT mice were subjected to CIH or normoxia condition for four weeks. B,C) Representative immunofluorescence staining of EZH2 in the distal femur section in (B) and analysis of cell number per mm^2^ tissue area (N. EZH2^+^ cells/ Ar) in (C). D,E) Representative immunofluorescence staining of UTX in the distal femur section (D) and analysis of cell number per mm^2^ tissue area (N. UTX^+^ cells/ Ar) in (E). F,G) Representative co‐immunofluorescence staining of H3K27me3 and OSX in distal femur section (F) and analysis of cell number per mm^2^ tissue area (N. H3K27me3^+^ OSX^+^ cells/ Ar) (G). H–K) Isolated BMSCs underwent osteoblast differentiation for 7 days and subsequently subjected to CIH or normoxia condition for 24 h. Then the treated osteoprogenitors were fixed and subjected to HIF1α and H3K27me3 (H), p19, and p21(I) immunostaining with quantification in (J) and (K), respectively. L–N) Isolated BMSCs underwent osteoblast differentiation for 7 days and subsequently subjected to CIH or normoxia condition for 24 h. Treated cells were harvested for western‐blot analysis of HIF1α, EZH2 and UTX in (L) and p21, p19, and H3K27me3 in (M). The isolated BMSCs were further treated with or without GSK‐J4 or GSK126 and harvested for western‐blot analysis of p21, p19 and H3K27me3 expression in (N). Quantifications of western‐blot were provided in Figures S5A,B and S6A (Supporting Information). *n* = 5–6 mice. Data were represented as mean ± SD. *p*‐values were determined by unpaired *t*‐tests or two‐way ANOVA.

Prior investigations have established that EZH2‐mediated trimethylation of histone H3 at lysine 27 (H3K27me3) serves as a vital epigenetic regulator influencing the initiation of cellular senescence in long bones.^[^ ^41^ ^]^ To explore the potential impact of CIH treatment on the EZH2‐H3K27me3 pathway in osteoprogenitors, we conducted an analysis of our scRNA‐Seq data, focusing on the expression of key enzymes involved in histone methylation and demethylation. Our results demonstrate that CIH treatment resulted in a decrease in the expression of histone methyltransferase EZH2 (**Figure** **3**A). In parallel, the levels of histone demethylases UTX and JMJD3 were found to be increased in mice exposed to CIH compared to those maintained under normoxia conditions (Figure 3A). Immunofluorescence staining corroborated a significant downregulation of EZH2 expression in the spongiosa of the femoral bone upon CIH treatment (Figure 3B,C). Consistently, the expression level of UTX was elevated in the CIH group compared with the Normoxia group (Figure 3D,E). Consequently, the population of OSX^+^ osteoprogenitors expressing H3K27me3 was significantly diminished in the metaphysis of CIH‐treated mice compared to those subjected to normoxia (Figure 3F,G).

To determine whether the CIH‐induced reduction of H3K27me3 is a direct consequence of treatment, we isolated bone marrow stromal cells (BMSCs) from the metaphysis of 4‐week‐old female mice and differentiated these cells into osteoprogenitors over a seven‐day period. The differentiated osteoprogenitors were then exposed to either normoxia or CIH for 24 h and subsequently processed for staining. Immunofluorescence analysis revealed that 24 h of CIH treatment led to a significant increase in HIF1α levels and a decrease in H3K27me3 levels (Figure 3H,J). Consistently, the expression of senescence markers p19 and p21 was also elevated (Figure 3I,K). HIF1α is a critical transcription factor involved in oxygen sensing, and previous studies have indicated that HIF1α overexpression results in the upregulation of a wide array of genes, including those associated with cellular senescence.^[^ ^42^ ^]^ To explore the oxygen‐sensing response across bone marrow‐derived cell populations under CIH, we analyzed the level of *Hif1a* in our single‐cell RNA sequencing dataset. *Hif1a* transcripts were detected in multiple clusters but displayed marked heterogeneity across cell types (Figure S3A, Supporting Information). Expression was most prominent in the mesenchymal lineage, particularly within osteoprogenitor clusters (Figure S4A, Supporting Information). This cell‐type–specific enrichment of *Hif1a* coincided with transcriptional signatures of senescence and impaired proliferation, suggesting that osteoprogenitors are transcriptionally primed to mount a stronger response to intermittent hypoxia. To confirm the effects of CIH on the downregulation of H3K27me3 and the upregulation of senescence‐related genes, we examined the expression of related proteins after in vitro CIH treatment. Immunoblot analysis demonstrated that osteoprogenitors exhibited elevated expression levels of HIF1α, along with increased EZH2 and reduced UTX following CIH treatment compared to normoxia treatment (Figure 3L) with quantification in Figure S5A (Supporting Information). Consistently, the level of senescence markers p19 and p21 was significantly upregulated, along with reduced trimethylated H3K27 after CIH treatment (Figure 3M) with quantification in Figure S5B (Supporting Information). Inhibition of the EZH2 using GSK126 resulted in decreased H3K27me3 levels and a significant increase in the expression of p19 and p21 in osteoprogenitors (Figure 3Q) with quantification in Figure S6A (Supporting Information). Notably, pharmacological inhibition of UTX by GSK‐J4 suppressed the expression of p19 and p21, in contrast to the pro‐senescence effect induced by EZH2 inhibition via GSK126, further confirming the role of H3K27me3 in repressing senescence‐related genes (Figure 3Q). Thus, the loss of Ezh2‐H3K27me3 is implicated in the promotion of cellular senescence by downregulating genes that facilitate cell proliferation and upregulating genes that induce cell cycle arrest.

### 2.4 Elevated H3K27me3 Alleviates CIH‐Induced Osteoprogenitor Senescence and Bone Loss

**Figure 4:**
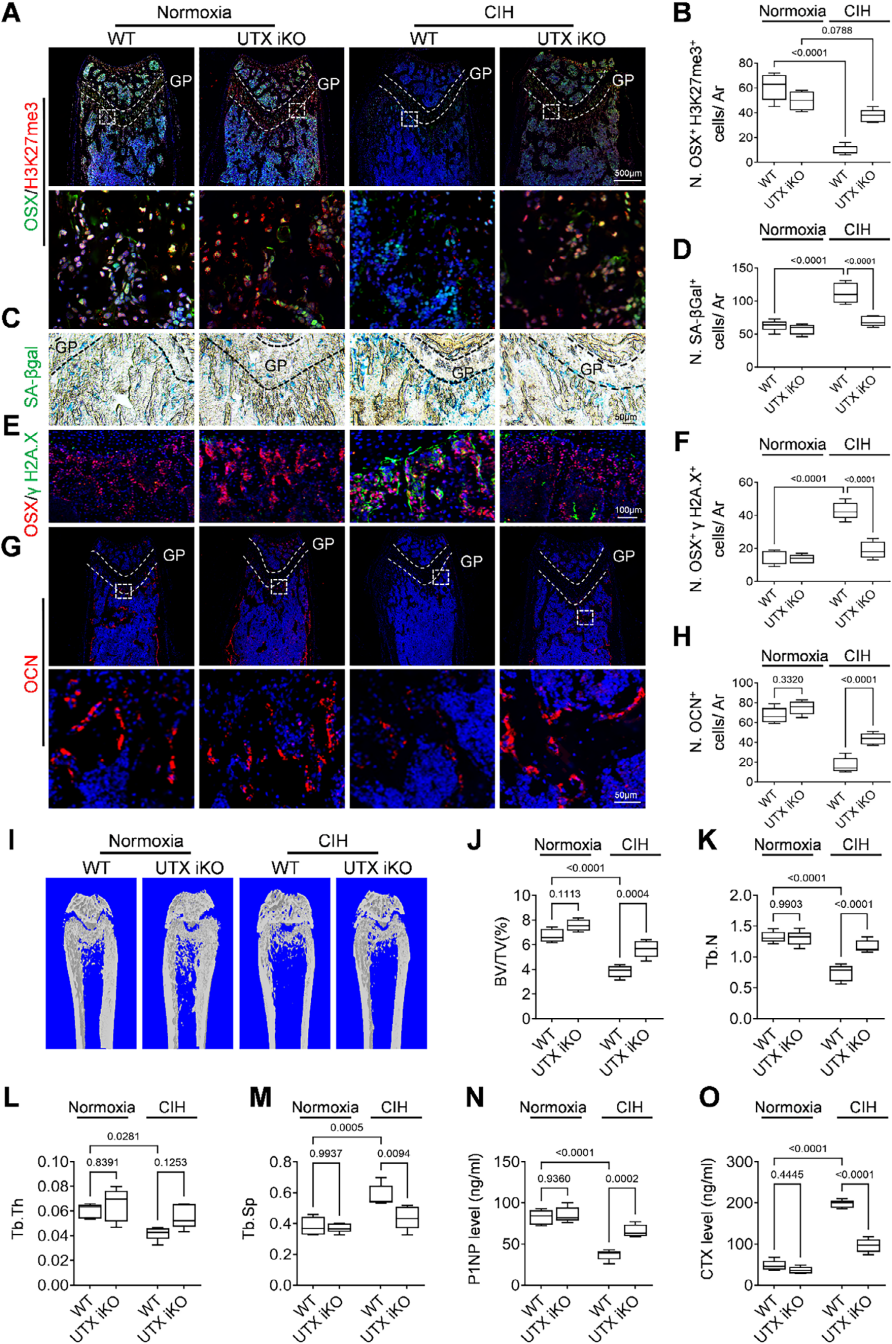
Elevated H3K27me3 alleviates CIH‐induced osteoprogenitor senescence and bone loss. Three‐week‐old female UTX iKO mice and WT (UTX floxed) mice were subjected to CIH or normoxia condition for four weeks. A,B) Representative co‐immunofluorescence staining of OSX and H3K27me3 in distal femur section in (A) and analysis of cell number per mm^2^ tissue area (N. OSX^+^ H3K27me3^+^ cells/ Ar) in (B). C,D) Representative images of SA‐βGal staining in the distal femur section in (C) and analysis of cell number per mm^2^ tissue area (N. SA‐βGal^+^ cells/ Ar) in (D). E,F) Representative co‐immunofluorescence staining of OSX and γ H2A.X in the distal femur section in (E) and analysis of cell number per mm^2^ tissue area (N. OSX^+^ γ H2A.X ^+^ cells/ Ar) in (F). G,H) Representative immunofluorescence staining of OCN in distal femur section in (G) and analysis of cell number per mm^2^ tissue area (N. OCN^+^ cells/ Ar) in (H). Representative micro‐CT images were shown in I) with quantitative analysis of BV/TV J), Tb. N K), Tb. Th L) and Tb. Sp M). N,O) The concentrations of P1NP and CTX were measured by ELISA. *n* = 5‐6 mice. Data were represented as mean ± SD. *p*‐values were determined by one‐way ANOVA.

We investigated the potential of elevated H3K27me3 levels to mitigate CIH‐induced senescence of osteoprogenitor cells and the associated impairment of osteogenesis. To this end, we utilized Osterix‐Cre^ERT2^::UTX^flox/flox^ mice (referred to as UTX iKO mice), in which *Utx* is inducibly deleted in OSX^+^ osteoprogenitor cells. Our findings revealed that CIH treatment consistently resulted in a loss of H3K27me3 in the femoral metaphysis of wild‐type (WT) mice; however, this effect was markedly alleviated in UTX iKO mice (**Figure** **4**A,B). To directly assess whether H3K27me3 modulation influences osteoprogenitor cell senescence under CIH, we examined senescence‐associated markers in femoral bone tissue. SA‐βgal staining revealed a significant reduction in senescent cells within the metaphyseal region of UTX iKO mice compared to CIH‐exposed controls (Figure 4C,D). Furthermore, immunofluorescence analysis showed that the number of γ H2A.X⁺ osteoprogenitors—marker of DNA damage and senescence—was markedly elevated in CIH‐treated mice but significantly reduced in UTX iKO mice (Figure 4E,F). The number of OCN^+^ osteoblasts in metaphysis was elevated in CIH‐treated UTX iKO mice compared to CIH‐treated WT mice, suggesting a preservation of osteogenesis in this area following H3K27me3 restoration (Figure 4G,H). CIH treatment induced a low‐bone‐mass phenotype in femoral metaphysis as described in the previous part (Figure 4I), characterized by reduced trabecular bone volume fraction (BV/TV), trabecular number (Tb.N), and trabecular thickness (Tb.Th), alongside an increase in trabecular separation (Tb.Sp) (Figure 4J–M). Notably, these alterations were largely rectified by UTX iKO. ELISA measurement of P1NP and CTX indicated increased bone formation activity along with decreased bone resorption (Figures 4N,O). Collectively, these results indicate that the elevation of H3K27me3 levels in the osteoprogenitors of the metaphysis of long bones, achieved through UTX ablation, effectively inhibits CIH‐induced cellular senescence in this region and mitigates the impairment of osteogenesis.

### 2.5 GSK‐J4 Attenuates CIH‐Induced Senescence Phenotype and Rescues CIH‐Impaired Bone Growth

**Figure 5:**
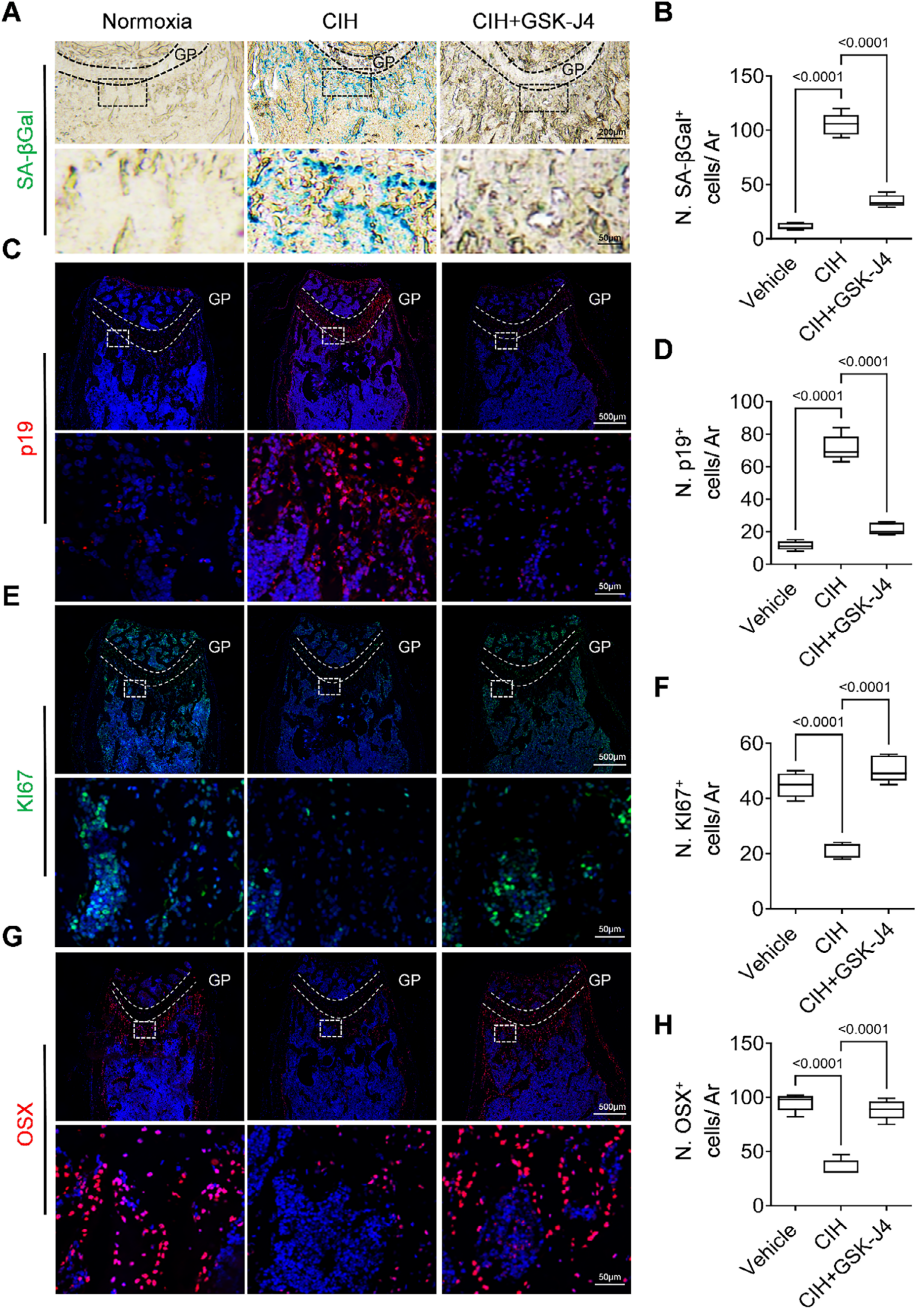
GSK‐J4 attenuates CIH‐induced senescence phenotype and promotes osteogenesis. Three‐week‐old female WT mice were subjected to CIH or normoxia condition for four weeks, and at the same time treated with GSK‐J4 at 10 mg kg^−1^ per day or vehicle. A‐B) Representative images of SA‐βGal staining in the distal femur section in (A) and analysis of cell number per mm^2^ tissue area (N. SA‐βGal^+^ cells/ Ar) in (B). C,D) Representative immunofluorescence staining of p19 in the distal femur section in (C) and analysis of cell number per mm^2^ tissue area (N. p19^+^ cells/ Ar) in (D). E,F) Representative immunofluorescence staining of Ki67 in the distal femur section in (E) and analysis of cell number per mm^2^ tissue area (N. Ki67^+^ cells/ Ar) in (F). G,H) Representative immunofluorescence staining of OSX in the distal femur section in (G) and analysis of cell number per mm^2^ tissue area (N. OSX ^+^ cells/ Ar) in (H). *n* = 5–6 mice. Data were represented as mean ± SD. p‐values were determined by one‐way ANOVA.

We assessed whether pharmacologically enhanced H3K27 methylation can rejuvenate senescent osteoprogenitors located in the metaphysis of long bones and thus promote bone formation. To this end, we co‐treated the CIH mice with the small molecule GSK‐J4, which promotes H3K27 methylation by inhibiting the demethylase activities of JMJD3 and UTX. CIH exposure led to a time‐dependent reduction in tail length and body weight compared to normoxic controls, consistent with impaired longitudinal growth. Importantly, co‐treatment with GSK‐J4 significantly rescued the reduction in tail length in CIH‐treated mice (Figure S7A, Supporting Information). GSK‐J4 treatment also slightly increased the body weight of CIH‐treated mice (Figure S7B, Supporting Information). At the experimental endpoint, we evaluated serum levels of alanine aminotransferase (ALT) and aspartate aminotransferase (AST) as indicators of liver function, and blood urea nitrogen (BUN) and creatinine (Cr) as markers of renal function. All values remained within the physiological range and showed no significant differences between treatment groups, indicating that GSK‐J4 does not cause detectable hepatotoxicity or nephrotoxicity at the administered dosage and duration (Figure S8A,B, Supporting Information). CIH treatment resulted in an increase in the number of SA‐βGal^+^ cells (**Figure** **5**A,B), an elevation in p19^+^ cell counts (Figure 5C,D), and a reduction in Ki67^+^ cell numbers (Figure 5E,F). Notably, co‐treatment with GSK‐J4 reversed these phenotypes. To further confirm the functional state of CIH‐induced senescent osteoprogenitors, we evaluated the expression of key senescence‐associated secretory phenotype (SASP) factors. ELISA analysis revealed that CIH significantly upregulated IL‐6, MMP13, and IGFBP3 in metaphysis bone tissue lysates, indicating the presence of a pro‐inflammatory and tissue‐degrading SASP phenotype. Treatment with GSK‐J4 suppressed these factors, consistent with a reversal of senescent signaling (Figure S9A–C, Supporting Information). Furthermore, the declines in osteogenesis induced by CIH were largely ameliorated by GSK‐J4 co‐treatment, as evidenced by immunofluorescence staining of OSX (Figure 5G,H) in femoral bone tissue sections. Therefore, increasing the levels of H3K27me3 by GSK‐J4 effectively mitigates the adverse effects of CIH on the developing skeleton.

**Figure 6:**
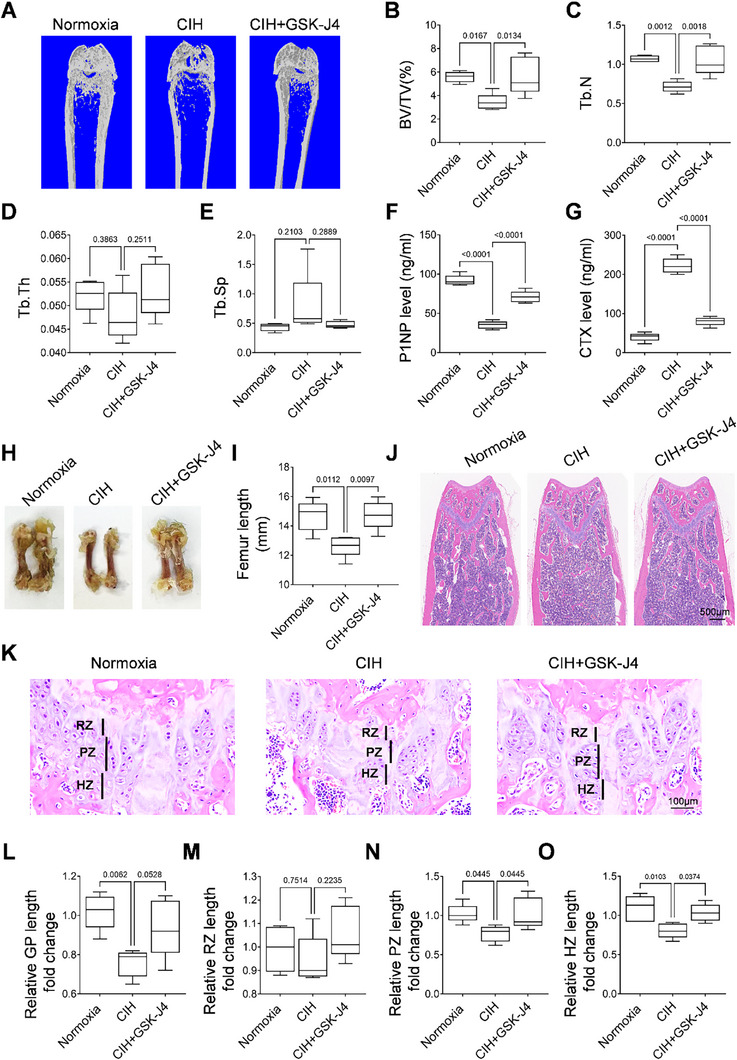
GSK‐J4 rescues CIH‐impaired bone growth and mineral acquisition. Three‐week‐old female WT mice were subjected to CIH or normoxia condition for four weeks, and at the same time, treated with GSK‐J4 at 10 mg kg^−1^ per day or vehicle. Representative micro‐CT images were shown in A) with quantitative analysis of BV/TV B), Tb. N C), Tb. Th D) and Tb. Sp E). F,G) The concentrations of P1NP and CTX were measured by ELISA. General view of the femurs was shown in H), and the length of the femurs were measured in I). J,K) H&E staining was performed to show tissue morphology. RZ, resting zone; PZ, proliferating zone; HZ, hypertrophic zone; GP, growth plate. The fold change of GP length, RZ length, PZ length, and HZ length were quantified in L–O). *n* = 5–6 mice. Data were represented as mean ± SD. *p*‐values were determined by one‐way ANOVA.

We subsequently investigated the efficacy of GSK‐J4 in preventing CIH‐induced bone loss. CIH exposure resulted in a low‐bone‐mass phenotype in the metaphysis of the femur (**Figure** **6**A), characterized by a reduced trabecular bone volume fraction (BV/TV), trabecular number (Tb.N), and trabecular thickness (Tb. Th), alongside an increase in trabecular separation (Tb.Sp) (Figure 6B–E). The alterations in these parameters were largely ameliorated by co‐treatment with GSK‐J4. To determine whether the protective effect of GSK‐J4 on bone growth is mediated directly through osteoprogenitor cells, we tested if GSK‐J4 treatment could further increase bone mass upon CIH treatment. In UTX iKO mice, GSK‐J4 treatment failed to further rescue CIH‐induced bone loss, indicating that its bone‐protective effect depends on UTX inhibition within osteoprogenitors (Figure S10A–E, Supporting Information). ELISA measurement of P1NP and CTX showed that the trend of decreasing bone formation activity and increasing bone resorption was reversed by GSK‐J4 treatment (Figure 6F,G). Notably, co‐treatment with GSK‐J4 effectively rescued the length of the femur impaired in the CIH‐treated group (Figure 6H,I). H–E staining of tissue sections demonstrated a reduced length of the distal femur growth plate in CIH‐treated mice compared to Normoxia controls; however, GSK‐J4 treatment largely reversed this detrimental effect (Figure 6J–L). The lengths of both the proliferative zone and hypertrophy zone were diminished in CIH‐treated mice, while the length of the resting zone remained unaffected (Figure 6M). Importantly, co‐treatment with GSK‐J4 improved the lengths of the proliferative and hypertrophy zones, which had been compromised by CIH (Figure 6N,O). Collectively, these data suggest that GSK‐J4 effectively protects growing bones from the adverse effects of CIH treatment on longitudinal bone growth and bone formation.

## 3 Discussion

OSAS in children is common and significantly affects the growth and overall health of adolescents. Severe yet untreated OSAS resulted in short stature and thus could have a lifetime impact on children.^[^ ^13^ ^]^ Although the harm of OSAS on bone health was long noted, few studies have explored how CIH affects bone growth, and the mechanism behind it remains largely unexplored. Thus, our study provides compelling evidence that CIH significantly impairs skeletal growth in young mice by disrupting bone formation via induction of osteoprogenitor senescence. Mechanistically, we showed that CIH induces osteoprogenitor senescence by upregulating *Hif1α* and subsequent modulation of epigenetic H3K27me3 mark. Finally, we showed that genetic and pharmacological blockage of osteoprogenitor senescence successfully restored osteogenesis activity, resulting in a rescue of CIH‐induced growth retardation and bone loss. Thus, our work unveils a novel cellular and molecular mechanism for the deleterious effects of OSAS on bone.

One of the most striking findings of our study is the significant reduction in tail length and femoral bone mass following CIH exposure. Notably, these effects were more severe in female mice compared to their male counterparts. Previous research has shown that hypoxia can disrupt endochondral ossification, a process critical for longitudinal bone growth, by inhibiting chondrocyte proliferation and differentiation.^[^ ^43^, ^44^, ^45^ ^]^ Our histological analyses corroborate these findings, as we observed a substantial decrease in growth plate height in CIH‐treated mice, with more pronounced reductions in the proliferative and hypertrophic zones in female mice. These findings suggest that CIH may differentially regulate chondrocyte activity in a sex‐dependent manner. Sex differences in bone response to hypoxia may be attributed to variations in hormonal regulation, particularly estrogen signaling. Estrogen plays a critical role in maintaining bone homeostasis by modulating osteoblast and osteoclast activity.^[^ ^46^, ^47^ ^]^ Serum hormone analysis indicated a significant decrease in estradiol levels in CIH‐exposed female mice, supporting the possibility that estrogen deficiency may amplify the deleterious skeletal effects of CIH. Estrogen is known to regulate endochondral ossification and osteoprogenitor differentiation, and its reduction may exacerbate growth plate dysfunction and bone loss under hypoxic stress. Given that estrogen levels in female mice are lower than in males at this developmental stage, CIH‐induced disruption of bone remodeling may be more severe in females due to reduced protective effects of estrogen.

Our single‐cell transcriptomic analysis revealed that while *Hif1α* was broadly induced by CIH, its expression and downstream transcriptional consequences were highly cell‐type specific. Osteoprogenitor populations exhibited the highest levels of Hif1a expression, along with co‐enrichment of senescence‐ and stress‐response pathways, whereas other lineages comparatively lower changes upon CIH treatment. This suggests that osteoprogenitors are uniquely sensitive to CIH‐induced hypoxia at the transcriptional level, which may explain why they undergo premature senescence and functional decline. These findings highlight a critical axis of transcriptional heterogeneity in hypoxic response, which underlies the selective vulnerability of osteoprogenitor cells under intermittent hypoxia condition.

Single‐cell RNA sequencing revealed significant enrichment of senescence‐related pathways in MSCs and osteoprogenitors following CIH treatment. Notably, the expression of cell cycle inhibitors p19 and p21 was markedly elevated, indicating an increased senescence burden in these populations. Tissue staining further confirmed the accumulation of senescent osteoprogenitors in CIH‐treated mice, as evidenced by increased senescence‐associated β‐galactosidase (SA‐βGal) staining and reduced Ki67 proliferation markers. Cellular senescence is known to impair tissue regeneration by inducing a pro‐inflammatory secretory phenotype (SASP) and disrupting progenitor cell function.^[^ ^48^ ^]^ The accumulation of senescent osteoprogenitors in CIH‐treated mice likely contributes to impaired osteoblast differentiation and decreased bone formation. Consistent with this notion, our gene expression analyses revealed a significant downregulation of osteogenic transcription factors in senescent MSCs. Future studies should investigate whether pharmacological clearance of senescent cells using senolytic drugs can mitigate CIH‐induced bone loss.

**Figure 7:**
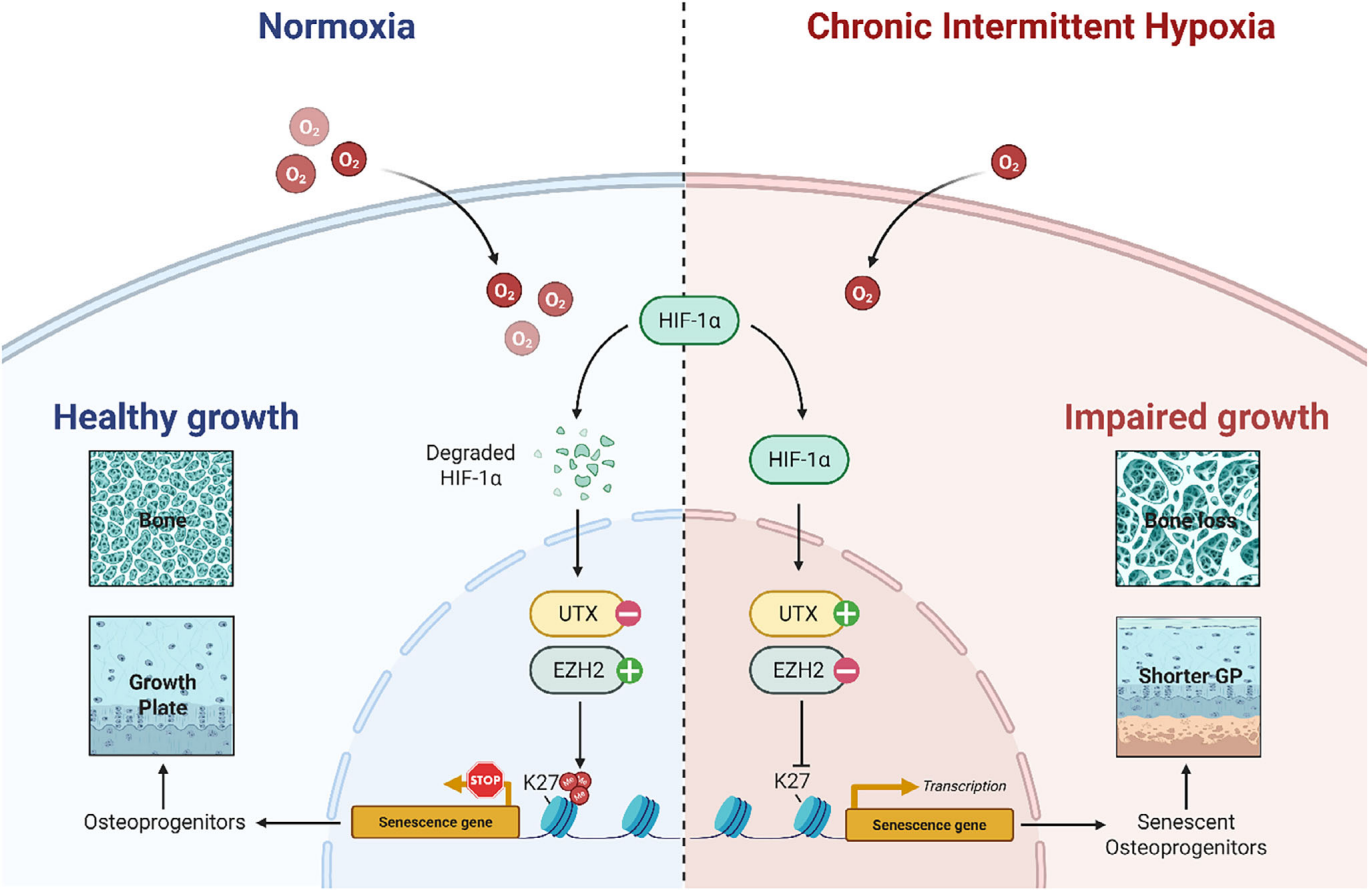
Scheme illustrating the mechanism of CIH‐induced Osteoprogenitor senescence in the metaphysis of growing bone.

Our study further elucidates an epigenetic mechanism linking CIH exposure to osteoprogenitor senescence (**Figure** **7**). We found that CIH downregulates the expression of Ezh2, a histone methyltransferase responsible for trimethylation of histone H3 at lysine 27 (H3K27me3), a key marker of transcriptional repression.^[^ ^49^ ^]^ Immunofluorescence staining confirmed a significant reduction in H3K27me3 levels in osteoprogenitors following CIH treatment. This epigenetic alteration was accompanied by increased expression of p19 and p21, suggesting that loss of Ezh2‐mediated transcriptional repression may drive osteoprogenitor senescence under hypoxic conditions. To establish a causal relationship, we used a genetic model in which UTX, a histone demethylase that opposes Ezh2 activity, was conditionally deleted in OSX^+^ osteoprogenitors. Notably, UTX ablation rescued CIH‐induced senescence and partially restored osteogenesis, demonstrating that histone methylation dynamics play a crucial role in regulating progenitor cell fate under hypoxia. Pharmacological inhibition of UTX using GSK‐J4 similarly attenuated CIH‐induced bone loss, further supporting the therapeutic potential of targeting histone modifications to counteract hypoxia‐mediated skeletal impairments. Our data revealed that EZH2 expression is robustly and consistently suppressed by hypoxic stress both in vivo and in vitro, supporting the concept that EZH2 downregulation is a conserved hallmark of hypoxia‐induced senescence. This finding aligns with previous reports that oxidative stress and p53 activation inhibit EZH2 expression to facilitate senescence‐associated transcriptional reprogramming.^[^ ^50^, ^51^ ^]^ These observations highlight that the imbalance between H3K27 methyltransferase and demethylase, for example EZH2 and UTX, is a core and conserved event in senescence induction under hypoxia.

Our findings have significant implications for clinical conditions associated with intermittent hypoxia, such as obstructive sleep apnea Syndrome (OSAS) and chronic lung diseases. OSAS is prevalent in children and adolescents and has been linked to growth retardation and skeletal abnormalities.^[^ ^52^, ^53^ ^]^ The mechanisms identified in our study suggest that OSA‐induced hypoxia may impair bone growth through similar pathways involving *Hif1α* activation and osteoprogenitor senescence. Future clinical studies should investigate whether biomarkers of senescence and epigenetic modifications are elevated in pediatric OSA patients with growth deficiencies. Additionally, our results highlight the therapeutic potential of targeting epigenetic regulators to mitigate hypoxia‐induced bone loss. The use of histone methyltransferase activators, such as GSK‐J4, represents a promising strategy to rejuvenate senescent osteoprogenitors and restore bone formation. Further preclinical studies are needed to assess the long‐term safety and efficacy of these approaches before clinical translation. The induction of SASP factors such as IL‐6, MMP13, and IGFBP3 in CIH‐exposed osteoprogenitors highlights the functional dimension of senescence beyond cell cycle arrest. SASP‐driven inflammation and matrix degradation are likely to exacerbate the impairment of bone formation and coupling of angiogenesis–osteogenesis under CIH conditions. The ability of GSK‐J4 to reduce both classical senescence markers and SASP factors suggests that targeting epigenetic regulators such as EZH2/H3K27me3 could alleviate not only intrinsic cellular senescence but also its paracrine effects on the bone microenvironment.

While our study provides novel insights into the epigenetic regulation of premature senescence in osteoprogenitor cells under chronic intermittent hypoxia (CIH), several limitations should be acknowledged. First, our experiments were conducted exclusively in young, growing mice, aiming to model pediatric OSAS‐related skeletal defects. As such, the long‐term consequences of early‐life CIH exposure on adult bone mass, remodeling, or fracture risk remain unclear. Second, our findings focus on stress‐induced senescence, and do not address how CIH may interact with age‐associated senescence programs or impact skeletal aging over time. Future studies employing longitudinal follow‐up, aged mouse models, or senescence‐tracking reporter systems will be essential to determine whether early senescent cell accumulation persists into adulthood and whether it predisposes individuals to impaired bone regeneration or osteoporosis later in life. Addressing these questions will further enhance the translational relevance of our findings to both pediatric and adult populations affected by OSAS. In summary, our study provides novel insights into the detrimental effects of CIH on bone growth and skeletal integrity. These findings underscore the need for targeted therapeutic strategies to counteract hypoxia‐induced skeletal impairments. Future research should focus on elucidating the long‐term effects of CIH on bone health and exploring potential interventions to prevent or reverse these deleterious changes.

## 4 Experimental Section

### Experimental Animals and Treatments

*C57BL/6J* mice (Strain NO. N000013), *Osterix‐Cre^ERT2^* mice (Cat. NO. NM‐KI‐232333), and *Utx^flox/flox^* mice (Strain NO. T000827) were purchased from Shanghai Model Organisms Center, Inc. and GemPharmatech (Nanjing, China), respectively. *Osterix‐Cre^ERT2^* mice were crossed with *Utx^flox/flox^* mice. The offspring were backcrossed with *Utx^flox/flox^* mice to generate *Osterix‐Cre^ERT2^; Utx^flox/flox^* mice (UTX iKO) and *Utx^flox/flox^* (WT) mice. To induce *CreER* activity, mice were injected at designed time points with tamoxifen (100 mg kg^−1^.B.W.) as described previously.^[^ ^54^ ^]^ The genotypes of the mice were determined by PCR analyses of genomic DNA extracted from a mouse tail/toe biopsy following protocols provided by the company. For GSK‐J4 administration, 3‐week‐old mice were treated with GSK‐J4 (10 mg kg^−1^ body weight, MedChemExpress, HY‐15648B) via daily intraperitoneal injection for 4 weeks. All animals were housed in the animal facility of Shanghai Jiaotong University. The experimental protocol was reviewed and approved by the Institutional Animal Care and Use Committee of Shanghai Jiaotong University with protocol number A2024434‐002.

### Chronic Intermittent Hypoxia (CIH) Exposure

Three‐week‐old mice were exposed to CIH in specialized chambers (Oxycycler model A84; BioSpherix, Redfield, NY, USA). These chambers were equipped with programmable solenoids and flow regulators that mimicked the fluctuations in arterial oxygen saturation seen in patients with obstructive sleep apnea (OSA). CIH exposure was carried out for 8 h per day, from 9:00 AM to 5:00 PM, over a period of 4 weeks. During each CIH session, the oxygen concentration gradually decreased from 21% to 5% over 150 s, hold at 5% for 10 s, followed by a rapid reoxygenation back to room air levels within the next 150 s. The normoxia group was placed in identical chambers with a constant oxygen level of 21%, while all other environmental conditions remained the same as the CIH group. In addition, drinking water was supplemented with a glucose and fructose mixture (42 g L^−1^, 55%/45%, w/w) throughout the CIH exposure period.

For cell experiments, the femurs and tibias of female mice were collected and placed in pre‐chilled PBS. Both bone ends were trimmed, and the marrow was flushed into a centrifuge tube using a 1 mL syringe with repeated PBS aspiration. After red blood cell lysis, the cells were cultured in 100 mm dishes with DMEM supplemented with 10% fetal bovine serum (Gibco), 100 U mL^−1^ streptomycin sulfate (Sigma‐Aldrich), and 100 U mL^−1^ penicillin (Sigma–Aldrich). The cells were incubated at 37 °C with 5% CO_2_ for 24 h, after which the medium was replaced to remove non‐adherent cells. Differentiation was induced by adding 0.1 mm dexamethasone, 10 mm β‐glycerophosphate, and 50 mm ascorbic acid to the culture medium. Medium was changed every 3 days for a total of 7 days. Subsequently, the differentiated cells were exposed to CIH conditions in identical chambers for 24 h. During this process, the oxygen fraction in the chamber was uniformly reduced from 21% to 1% over a 15 min period, and maintained at 1% for 10 min, then uniformly increased to 21% over a 15 min period and maintained at 21% for 1 min. The whole process was cyclical, with a constant balance of 5% CO_2_ and nitrogen. For immunofluorescence staining, treated cells were fixed and incubated with primary antibodies to HIF‐1α (Cell Signaling Technology, 36169, 1:400), H3K27me3 (Cell Signaling Technology, 9733, 1:800), p19 (Proteintech, 10272‐2‐AP, 1:100), and p21 (Proteintech, 28248‐1‐AP, 1:200).

### Bone Phenotype Analysis

For micro‐CT analysis, femoral bone from mice was harvested, cleaned of soft tissue, fixed overnight in 10% formalin at 4 °C, and scanned using a high‐resolution µCT scanner (Skyscan 1175, Bruker MicroCT, Belgium). The scanner settings were 65 kV, 153 µA, and a resolution of 9.0 µm/pixel. Image reconstruction was performed using NRecon (version 1.6), and data analysis was conducted with CTAn (version 1.9) and CTVol (version 2.0) software. The trabecular bone parameters (100 layers) in the metaphysis were measured, including BV/TV, Tb. Th, Tb. N, and Tb. Sp, as previously described.^[^ ^30^ ^]^

### Immunohistochemistry and Immunofluorescence Staining of Bone Tissue Sections

For histochemistry analysis, the femur was dissected and fixed in 10% formalin for 24 h, decalcified in 0.5 m ethylenediaminetetraacetic acid (EDTA, pH 7.6) at 4 °C with constant shaking for seven days, dehydrated, and embedded in paraffin. 4‐µm‐thick longitudinally oriented sections of distal femoral bone, including the metaphysis and diaphysis, were processed for hematoxylin‐eosin staining. All sections were observed and imaged using an Olympus BX51 microscope. For frozen sections, after decalcification, the bones were immersed in 20% sucrose supplemented with 2% polyvinylpyrrolidone solution for 24 h. Finally, the tissues were embedded in O.C.T, and 10‐µm‐thick, longitudinally oriented bone sections were collected for staining. Senescent cells were detected using a senescence βGal staining kit according to the manufacturer's instructions (Cell Signaling Technology, Danvers, MA). For immunofluorescence staining, the sections with primary antibodies were incubated to H3K27me3 (Cell Signaling Technology, 9733, 1:800), Ki67 (Abcam, ab15580, 1:200), osterix (Abcam, ab22552, 1:200), osteocalcin (Takara, M188, 1:200), p21 (Proteintech, 28248‐1‐AP, 1:200), p19 (Proteintech, 10272‐2‐AP, 1:100), UTX (Cell Signaling Technology, 33510, 1:300), Ezh2 (Cell Signaling Technology, 5246, 1:300), HIF‐1α (Cell Signaling Technology, 36169, 1:400) overnight at 4 °C, followed by incubation with Alexa Fluor 488, or Alexa Fluor 594 secondary antibodies (Jackson ImmunoResearch). Nuclei were counterstained and mounted with mounting medium with DAPI (Vector Laboratories, H‐1200) and observed under a Zeiss LSM780 confocal microscope.

### ELISA

Blood serum was collected by cardiac puncture before sacrifice and stored at −80 °C until use. N‐terminal propeptide of type I procollagen (P1NP) was measured using P1NP ELISA kit (E‐EL‐M0233, Elabscience) and β‐CTx ELISA kit (E‐EL‐M0372, Elabscience) as described by the company. For SASP factor measurement, the mice femoral bone tissue was collected and processed for ELISA measurement as described by the manufacturer. IL‐6 ELISA kit (E‐EL‐M0044, Elabscience), MMP13 ELISA kit (E‐EL‐M0076, Elabscience), and IGFBP3 ELISA kit (E‐EL‐M3075, Elabscience) were used for the measurement of SASP factors.

### Western Blot Analysis

Western blot analysis was performed as previously described.^[^ ^55^ ^]^ Briefly, the mouse femur was dissected, and adherent muscles and periosteum around the bone were removed. The epiphysis was removed, and only the 3‐mm‐long metaphyseal regions from the growth plate were crushed in ice‐cold RIPA lysis buffer with a mortar and pestle. The total protein from the tissue extract was subjected to SDS‐PAGE and blotted onto a polyvinylidene difluoride (Bio‐Rad Laboratories) membrane. The membrane was incubated with antibodies against p19 (Proteintech, 10272‐2‐AP, 1:1000), p21 (Proteintech, 28248‐1‐AP, 1:1000), H3K27me3 (Cell Signaling Technology, 9733, 1:1000), and β‐Actin (ABclonal, AC026, 1:100000), respectively, and visualized by enhanced chemiluminescence (ECL Kit; Amersham Biosciences).

### Single‐Cell Transcriptome Sequencing and Bioinformatics Analysis

For the preparation of a single cell suspension from the femoral metaphysis, mice femoral bone was dissected and cleared free of soft tissue. The epiphysis was removed gently by forceps, and only the 3‐mm‐long metaphyseal regions from the growth plate were crushed with a mortar and pestle in DMEM medium. The crushed tissue and released cells were digested in 2 mg mL^−1^ type I collagenase at 37 °C with constant shaking for 30 min, filtered through a 70 µm cell strainer, washed once with ice‐cold PBS, and resuspended in DMEM medium. Sequencing libraries were generated following a comprehensive transcriptome sequencing and single‐cell gene‐expression profiling protocol as previously reported. Briefly, Single‐cell RNA‐Seq libraries were generated using the SeekOne Digital Droplet Single Cell 3′ library preparation kit (SeekGene, Beijing, China) and sequenced on an Illumina NovaSeq X Plus with PE150 read length. AddModuleScore was used to calculate the enrichment score of each cluster on MSigDB genesets. ScRNA‐seq data had been deposited at the Gene Expression Omnibus (GEO) with the accession number GSE304165.

### Statistics

Data are presented as mean ± standard deviation (SD) of the mean. Unpaired, 2‐tailed Student *t*‐tests were used for comparisons between two groups. For multiple comparisons, one‐way or two‐way analysis of variance (ANOVA) with a Bonferroni post hoc test was used. All data were normally distributed and had similar variation between groups. Statistical analysis was performed using GraphPad Prism version7.04, software (GraphPad Software, Inc., San Diego, CA.). *p* <0.05 was deemed significant.

### Data Availability

ScRNA‐seq data had been deposited at the Gene Expression Omnibus (GEO) with the accession number GSE304165 and was publicly available. All data that supports the findings of this study are available within the article and Supplementary Files or from the corresponding author upon reasonable request.

## Author Contributions

X.L., P.Z., Z.S., and Y.F. contributed equally to this work. X.L. designed the experiments and drafted the manuscript; Z.S. and P.Z. carried out most of the experiments; Z.Z., S.P., X.L., Y.W., J.H., and Y.F. helped with some experiments; X.L. and P.Z. proofread the manuscript; X.L. and P.Z. supervised the experiments and analyzed the results.

## Conflict of Interest

The authors declare no conflict of interest.

## Supporting information



Supporting Information

## Data Availability

The data that support the findings of this study are available from the corresponding author upon reasonable request.
