# Supplementary material for: Epigenetic Targeting of Senescent Cells Prevents the Deleterious Effects of Obstructive Sleep Apnea on Growing Skeleton
Source: Adv Sci (Weinh). 2025 Dec 12;13(4):e02697. doi: 10.1002/advs.202502697 (PMC12822469; doi:10.1002/advs.202502697)
Supplement: Supplementary file 1 — Supporting Information [file ADVS-13-e02697-s001.docx]

**
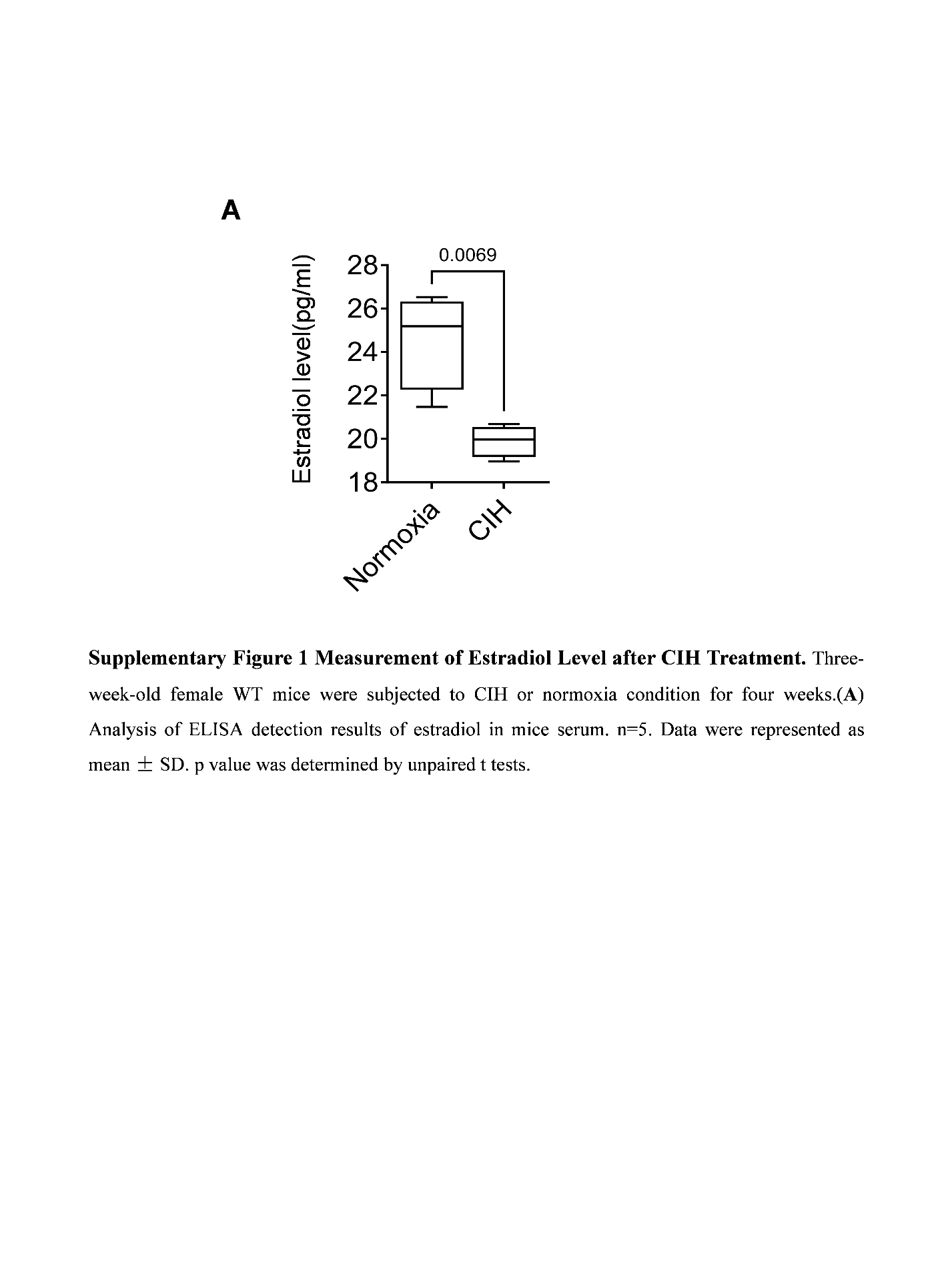
**

**Supplementary Figure 1 Measurement of Estradiol Level after CIH Treatment.** Three-week-old female WT mice were subjected to CIH or normoxia condition for four weeks. (**A**) Analysis of ELISA detection results of estradiol in mice serum. n=5. Data were represented as mean ± SD. p value was determined by unpaired t tests.

**
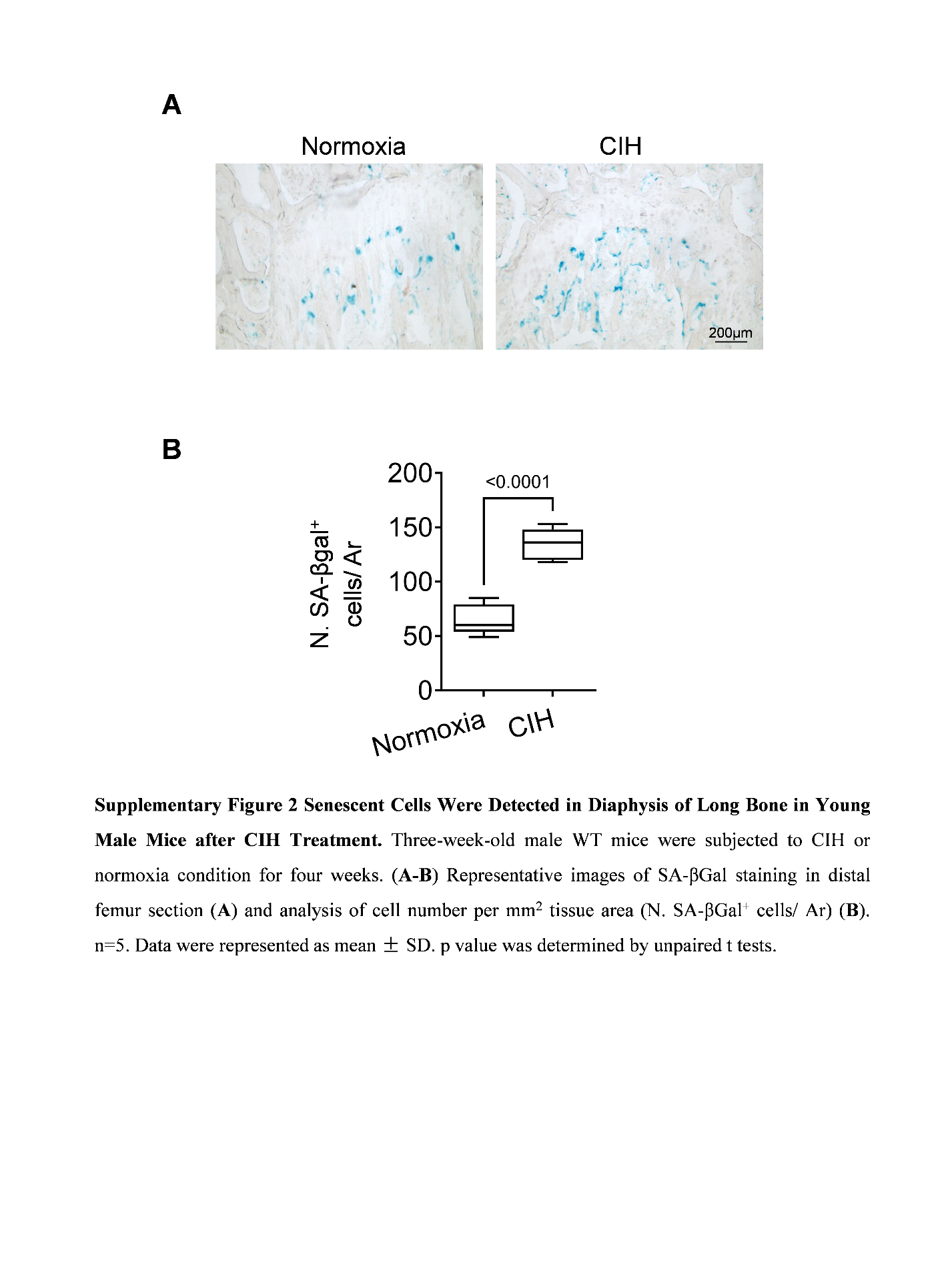
**

**Supplementary Figure 2 Senescent Cells Were Detected in Diaphysis of Long Bone in Young Male Mice after CIH Treatment.** Three-week-old male WT mice were subjected to CIH or normoxia condition for four weeks. (**A-B**) Representative images of SA-βGal staining in distal femur section (**A**) and analysis of cell number per mm^2^ tissue area (N. SA-βGal^+^ cells/ Ar) (**B**). n=5. Data were represented as mean ± SD. p value was determined by unpaired t tests.

**
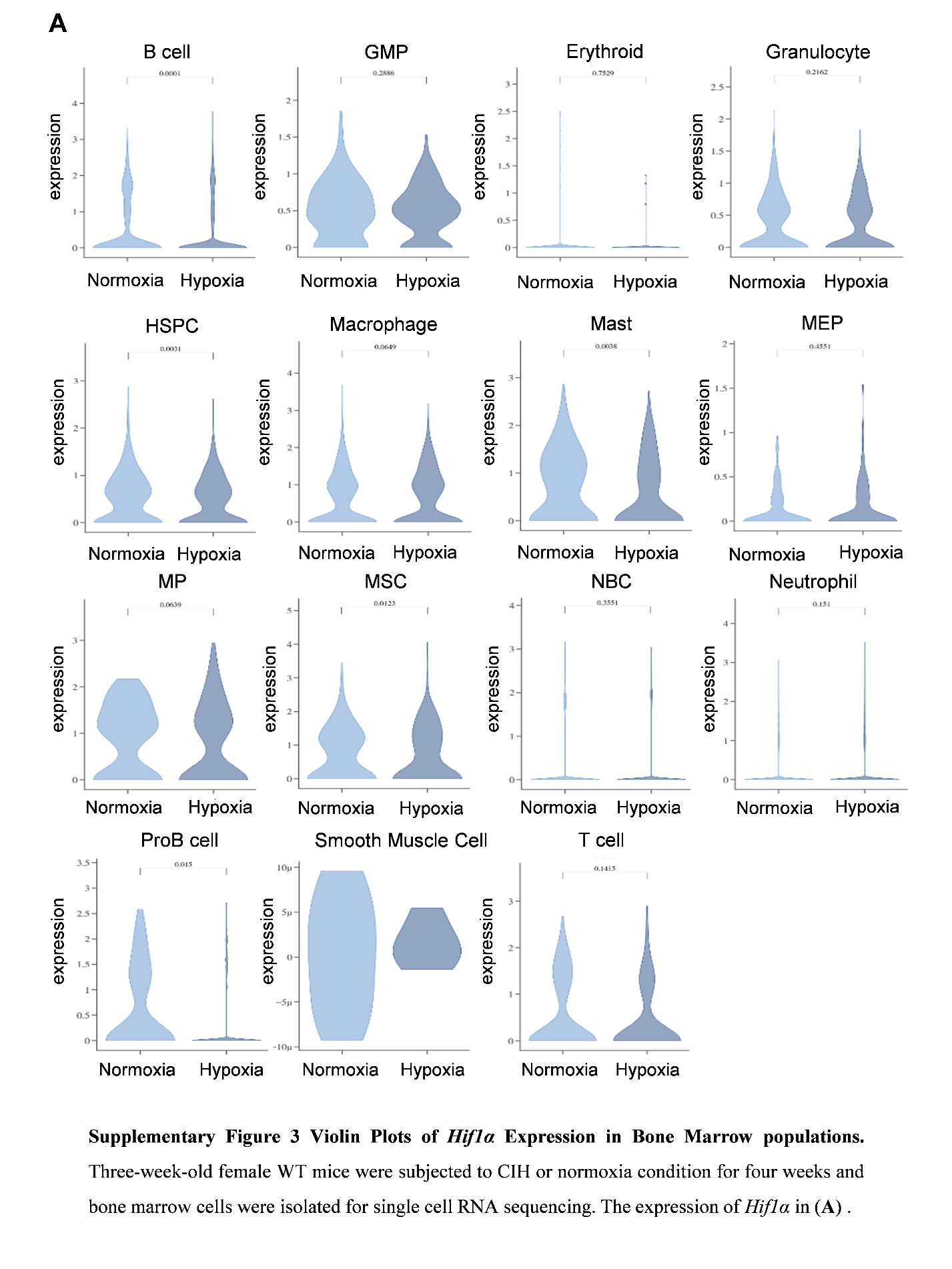
**

**Supplementary Figure 3 Violin Plots of *Hif1α* Expression in Bone Marrow populations.** Three-week-old female WT mice were subjected to CIH or normoxia condition for four weeks and bone marrow cells were isolated for single cell RNA sequencing. The expression of *Hif1α* in (**A**).

**
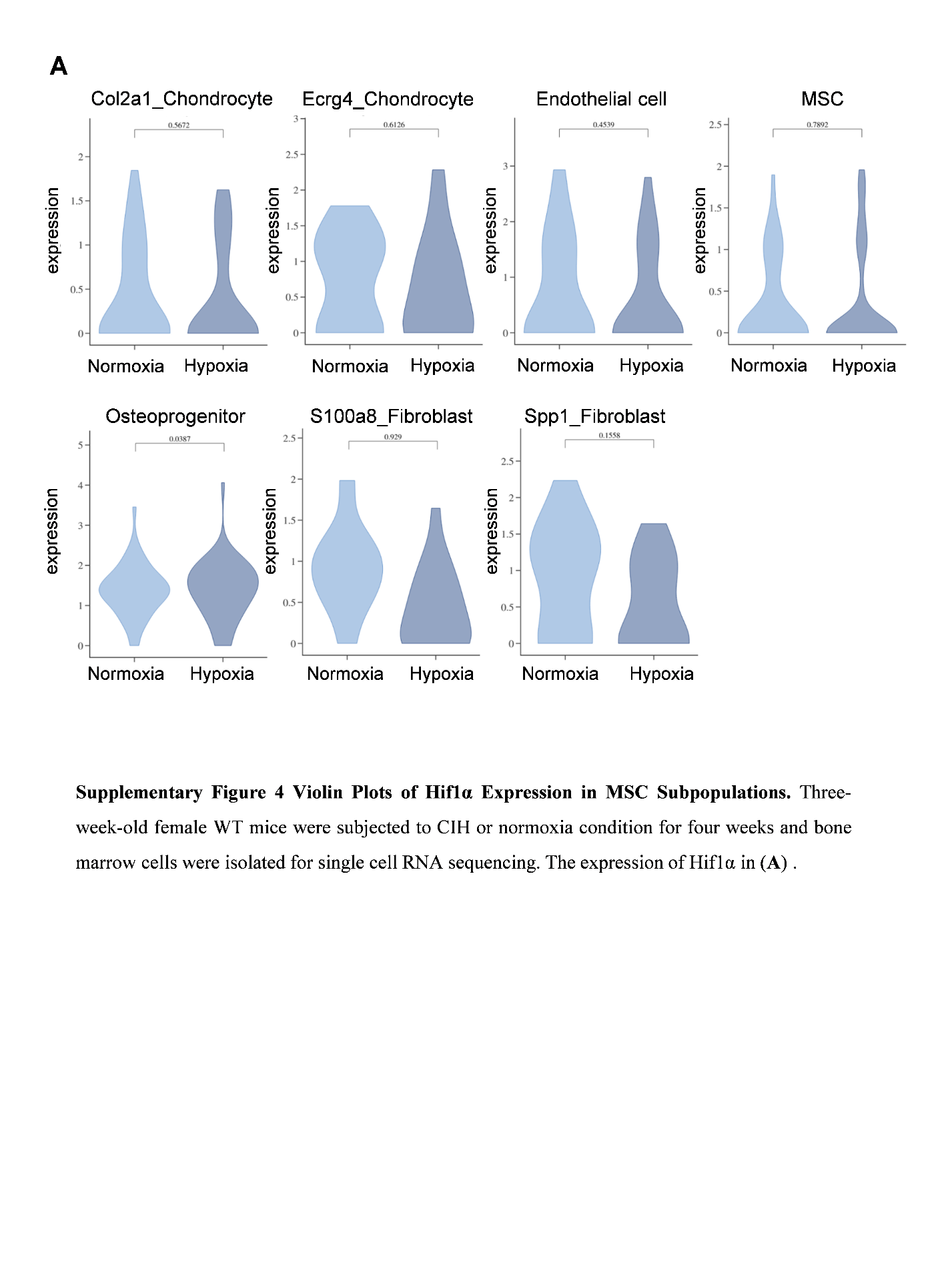
**

**Supplementary Figure 4 Violin Plots of Hif1α Expression in MSC Subpopulations.** Three-week-old female WT mice were subjected to CIH or normoxia condition for four weeks and bone marrow cells were isolated for single cell RNA sequencing. The expression of Hif1α in (**A**).

**
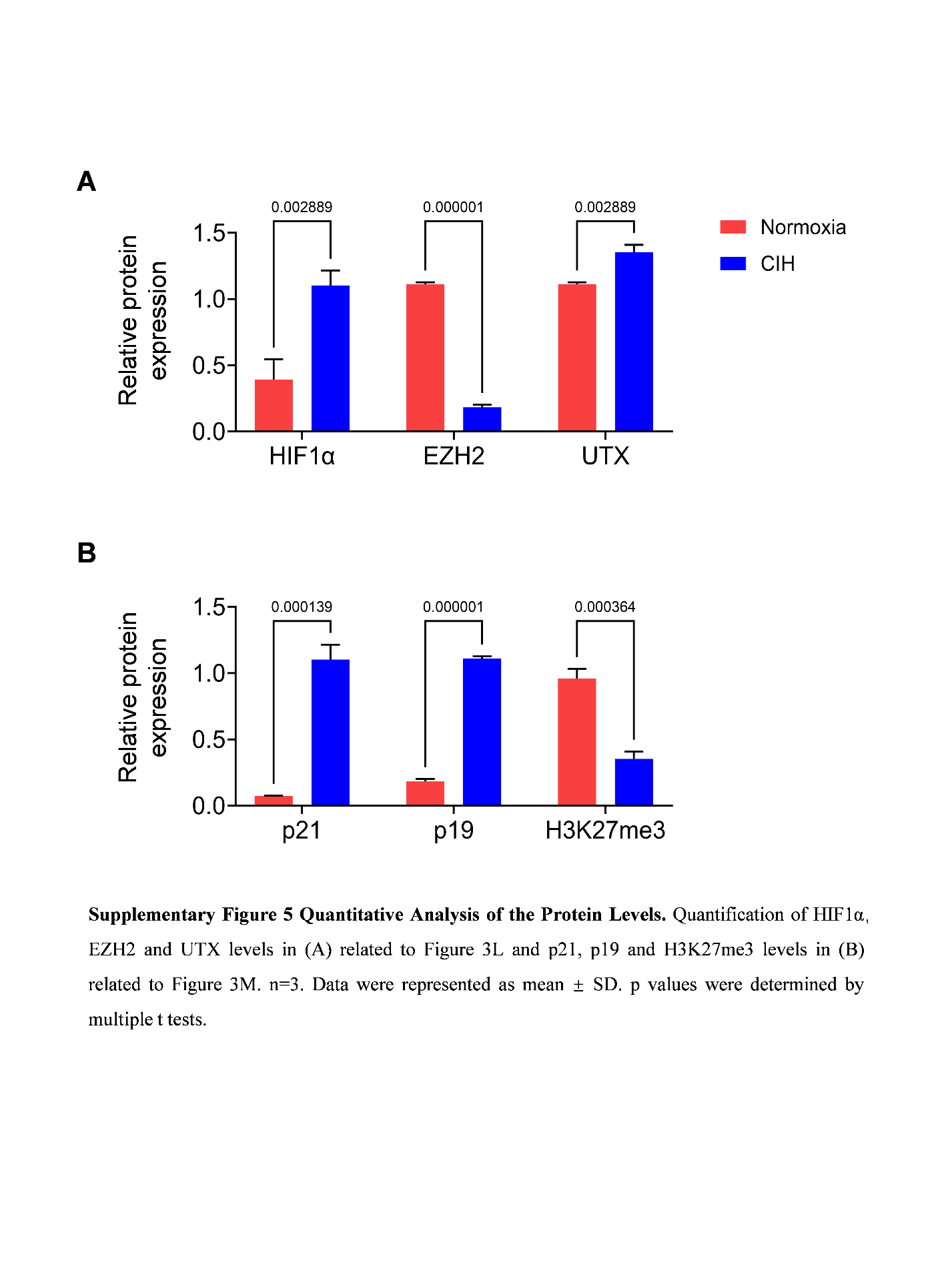
**

**Supplementary Figure 5 Quantitative Analysis of the Protein Levels.** Quantification of HIF1α，EZH2 and UTX levels in (**A**) related to Figure 3L and p21, p19 and H3K27me3 levels in (**B**) related to Figure 3M. n=3. Data were represented as mean ± SD. p values were determined by multiple t tests.

**
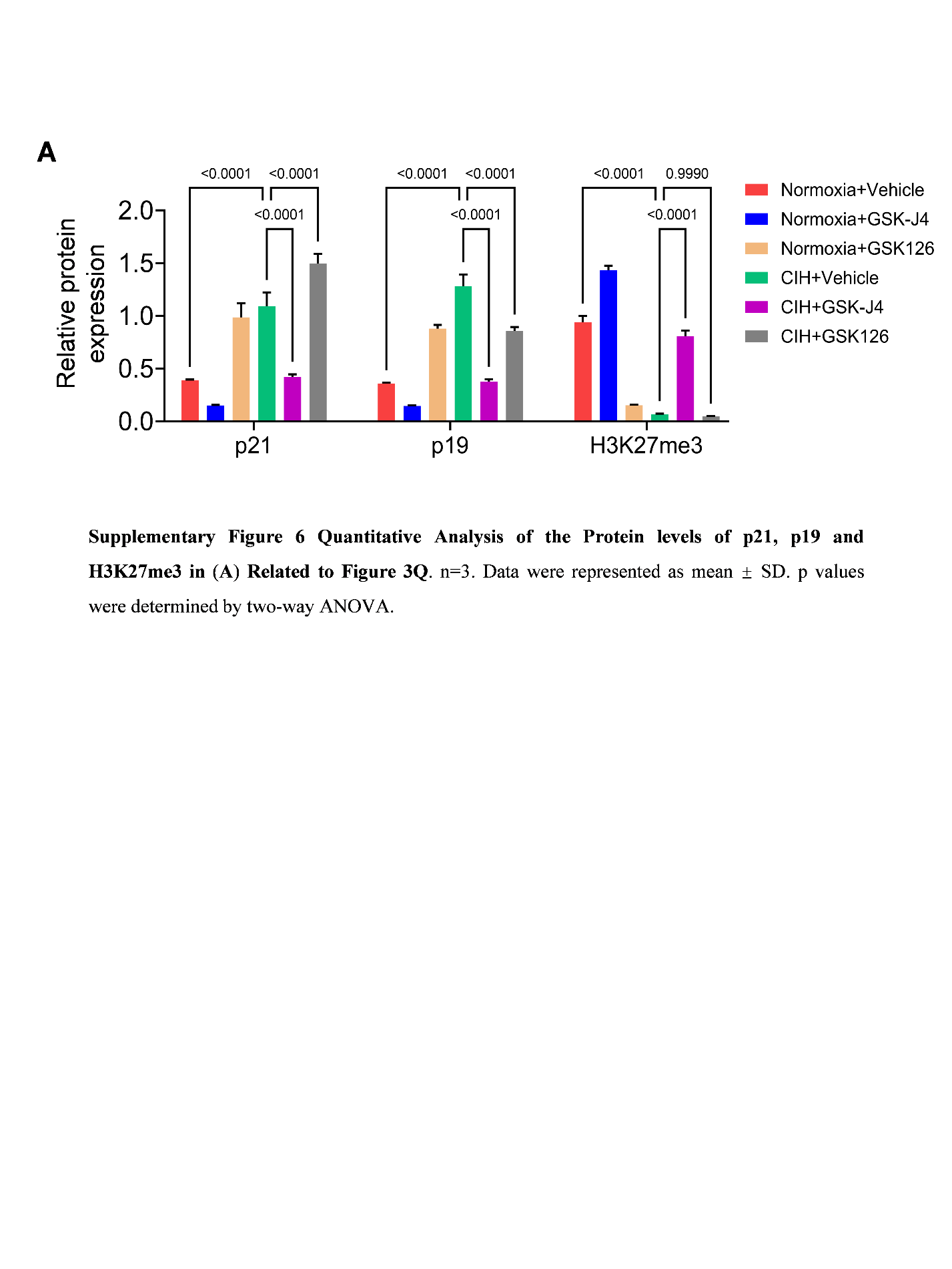
**

**Supplementary Figure 6 Quantitative Analysis of the Protein levels of p21, p19 and H3K27me3 in (A) Related to Figure 3Q.** n=3. Data were represented as mean ± SD. p values were determined by two-way ANOVA.

**
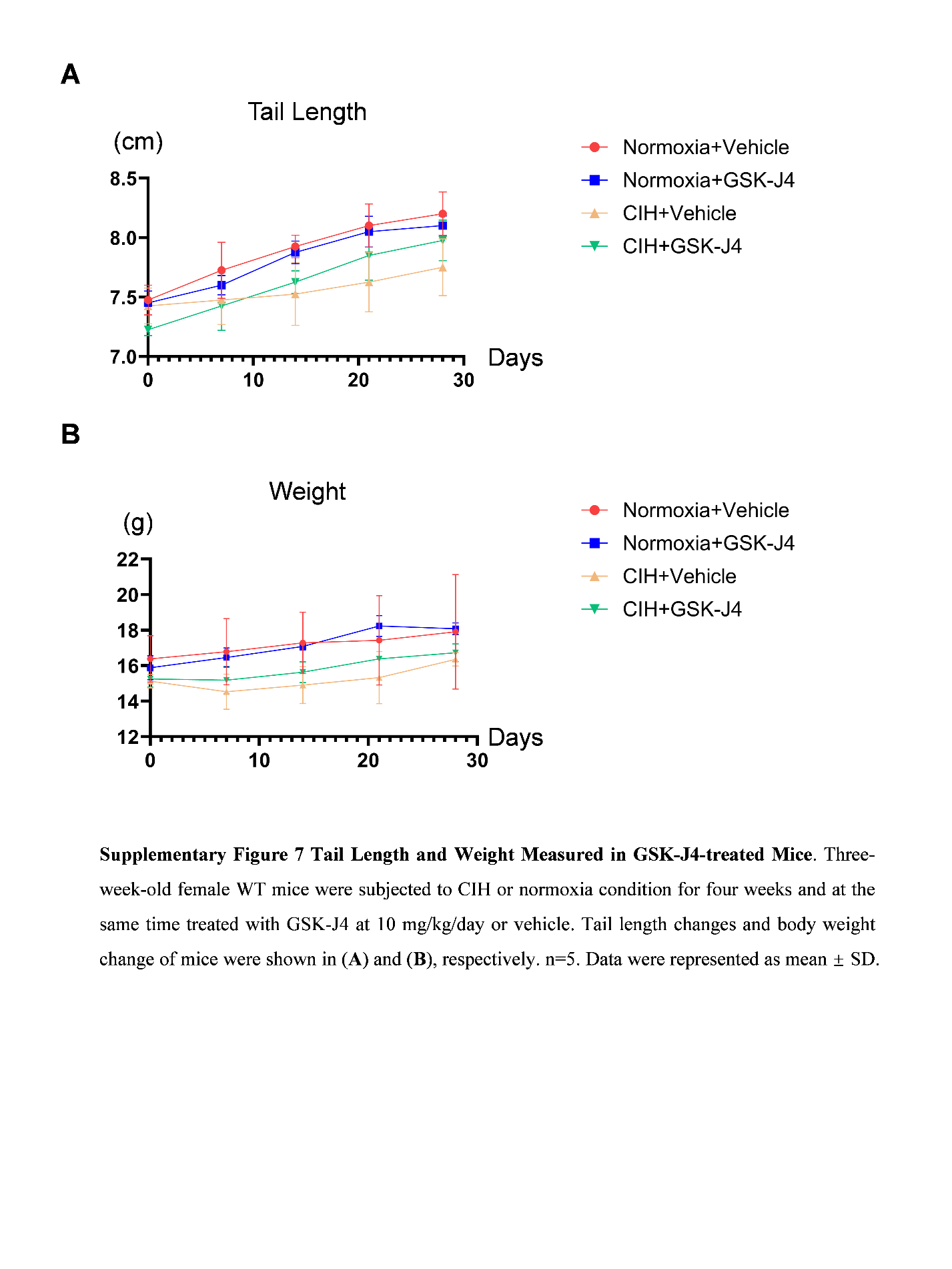
**

**Supplementary Figure 7 Tail Length and Weight Measured in GSK-J4-treated Mice.** Three-week-old female WT mice were subjected to CIH or normoxia condition for four weeks and at the same time treated with GSK-J4 at 10 mg/kg/day or vehicle. Tail length changes and body weight change of mice were shown in (**A**) and (**B**), respectively. n=5. Data were represented as mean ± SD.

**
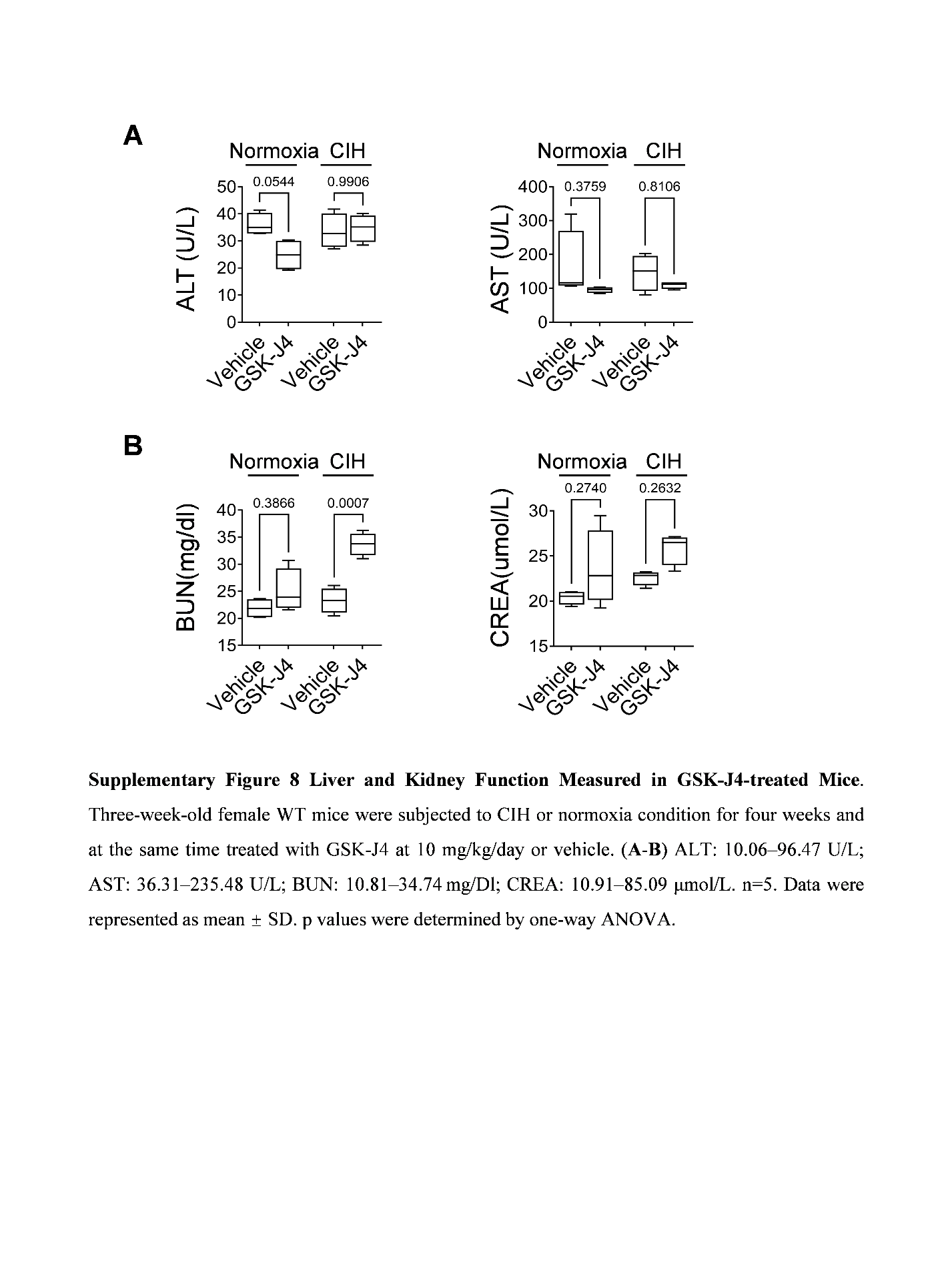
**

**Supplementary Figure 8 Liver and Kidney Function Measured in GSK-J4-treated Mice.** Three-week-old female WT mice were subjected to CIH or normoxia condition for four weeks and at the same time treated with GSK-J4 at 10 mg/kg/day or vehicle. (**A-B**) ALT: 10.06–96.47 U/L; AST: 36.31–235.48 U/L; BUN: 10.81–34.74 mg/Dl; CREA: 10.91–85.09 μmol/L. n=5. Data were represented as mean ± SD. p values were determined by one-way ANOVA.

**
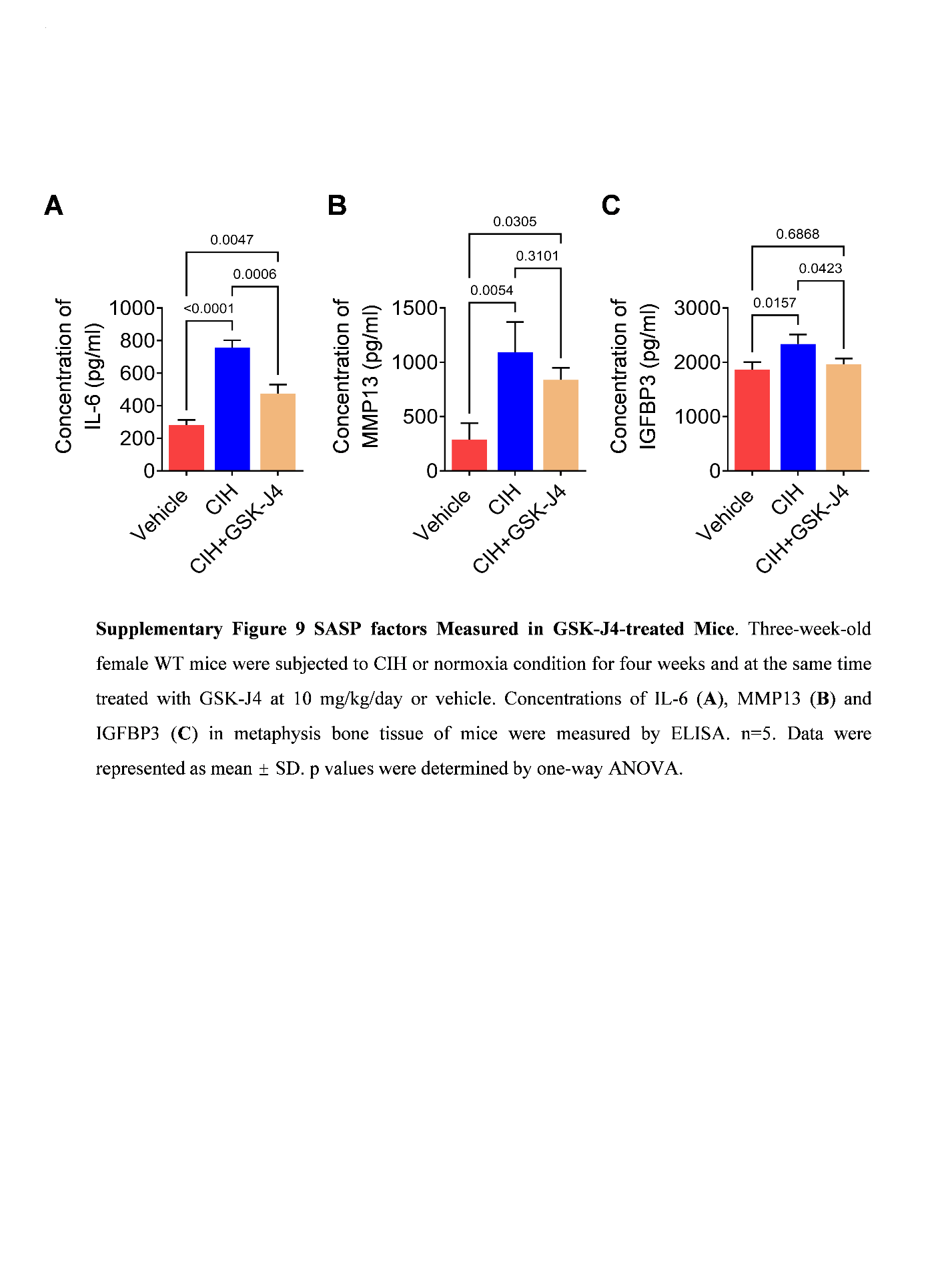
**

**Supplementary Figure 9 SASP factors Measured in GSK-J4-treated Mice.** Three-week-old female WT mice were subjected to CIH or normoxia condition for four weeks and at the same time treated with GSK-J4 at 10 mg/kg/day or vehicle. Concentrations of IL-6 (**A**), MMP13 (**B**) and IGFBP3 (**C**) in metaphysis bone tissue of mice were measured by ELISA. n=5. Data were represented as mean ± SD. p values were determined by one-way ANOVA.

**
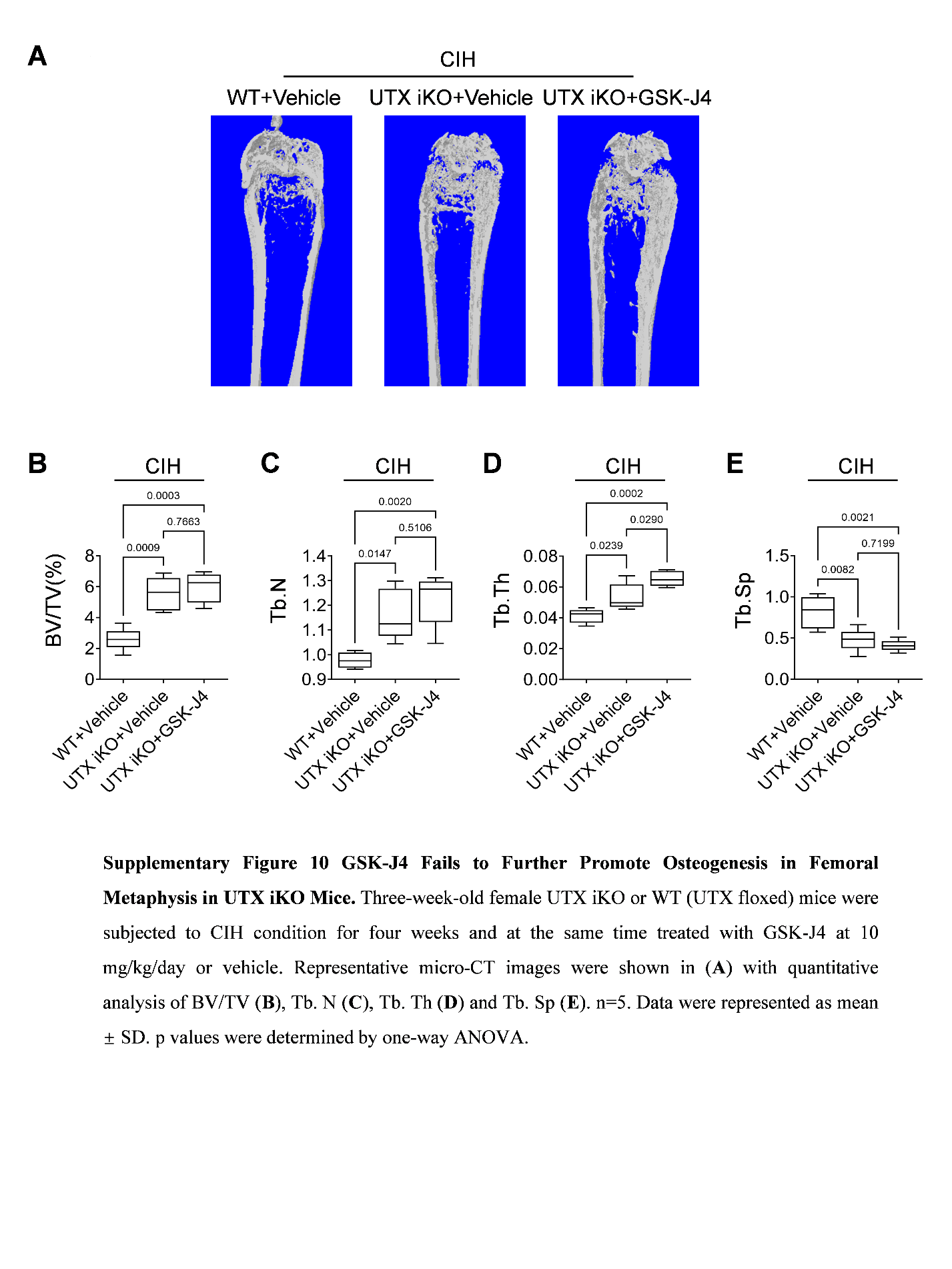
**

**Supplementary Figure 10 GSK-J4 Fails to Further Promote Osteogenesis in Femoral Metaphysis in UTX iKO Mice.** Three-week-old female UTX iKO or WT (UTX floxed) mice were subjected to CIH condition for four weeks and at the same time treated with GSK-J4 at 10 mg/kg/day or vehicle. Representative micro-CT images were shown in (**A**) with quantitative analysis of BV/TV (**B**), Tb. N (**C**), Tb. Th (**D**) and Tb. Sp (**E**). n=5. Data were represented as mean ± SD. p values were determined by one-way ANOVA.

**References**

[1] A. V. Benjafield, N. T. Ayas, P. R. Eastwood, R. Heinzer, M. S. M. Ip, M. J. Morrell, C. M. Nunez, S. R. Patel, T. Penzel, J. Pepin, P. E. Peppard, S. Sinha, S. Tufik, K. Valentine, A. Malhotra, *Lancet Respir Med* **2019**, *7*, 687.

[2] R. Heinzer, H. Marti-Soler, J. Haba-Rubio, *Lancet Respir Med* **2016**, *4*, e5.

[3] L. Grote, *Lancet Respir Med* **2019**, *7*, 645.

[4] S. Garbarino, N. Magnavita, A. Sanna, N. L. Bragazzi, *Lancet Respir Med* **2020**, *8*, e1.

[5] L. Pereira, E. Song, C. M. Stefani, C. Flores-Mir, G. De Luca Canto, C. Pacheco-Pereira, *Sleep Med Rev* **2024**, *80*, 102040.

[6] A. C. Bitners, R. Arens, *Lung* **2020**, *198*, 257.

[7] H. Xie, W. Yung, *Acta Pharmacol Sin* **2012**, *33*, 5.

[8] C. Arnaud, E. Billoir, A. F. de Melo Junior, S. A. Pereira, K. D. O'Halloran, E. C. Monteiro, *J Physiol* **2023**, *601*, 5553.

[9] K. Zhang, D. Ma, Y. Wu, Z. Xu, *Nat Sci Sleep* **2024**, *16*, 2029.

[10] S. L. Blunden, D. W. Beebe, *Sleep Med Rev* **2006**, *10*, 109.

[11] L. Zhang, L. Jin, J. Guo, K. Bao, J. Hu, Y. Zhang, Z. Hou, L. Zhang, *Front Endocrinol (Lausanne)* **2020**, *11*, 582670.

[12] Y. Chen, S. Weng, Y. Shen, C. Chou, C. Yang, J. Wang, K. Tien, *J Clin Endocrinol Metab* **2014**, *99*, 2441.

[13] T. V. Serebrovskaya, L. Xi, *Front Pediatr* **2015**, *3*, 44.

[14] R. Farahani, A. Kanaan, O. Gavrialov, S. Brunnert, R. M. Douglas, P. Morcillo, G. G. Haddad, *Pediatr Pulmonol* **2008**, *43*, 20.

[15] R. Rizzoli, M. L. Bianchi, M. Garabedian, H. A. Mckay, L. A. Moreno, *Bone* **2010**, *46*, 294.

[16] A. E. Schettler, E. M. Gustafson, *J Am Acad Nurse Pract* **2004**, *16*, 274.

[17] S. E. Mccormack, D. L. Cousminer, A. Chesi, J. A. Mitchell, S. M. Roy, H. J. Kalkwarf, J. M. Lappe, V. Gilsanz, S. E. Oberfield, J. A. Shepherd, K. K. Winer, A. Kelly, S. F. A. Grant, B. S. Zemel, *JAMA PEDIATRICS* **2017**, *171*, 9.

[18] R. Florencio-Silva, G. R. D. S. Sasso, E. Sasso-Cerri, M. J. Simoes, P. S. Cerri, *Biomed Res Int* **2015**, *2015*, 421746.

[19] M. Hirao, N. Tamai, N. Tsumaki, H. Yoshikawa, A. Myoui, *J Biol Chem* **2006**, *281*, 31079.

[20] C. E. Yellowley, D. C. Genetos, *Curr Osteoporos Rep* **2019**, *17*, 26.

[21] Q. Qin, Y. Liu, Z. Yang, M. Aimaijiang, R. Ma, Y. Yang, Y. Zhang, Y. Zhou, *Int J Mol Sci* **2022**, *23*.

[22] J. Lee, S. Bae, J. Jeong, S. Kim, K. Kim, *Exp Mol Med* **2004**, *36*, 1.

[23] R. Belapurkar, M. Pfisterer, J. Dreute, S. Werner, S. Zukunft, I. Fleming, M. Kracht, M. L. Schmitz, *Cell Death Dis* **2023**, *14*, 477.

[24] L. Chen, A. Endler, F. Shibasaki, *Exp Mol Med* **2009**, *41*, 849.

[25] C. T. Taylor, C. C. Scholz, *Nat Rev Nephrol* **2022**, *18*, 573.

[26] W. Chen, P. Wu, F. Yu, G. Luo, L. Qing, J. Tang, *Cells* **2022**, *11*.

[27] X. Liu, M. Wan, *Int Rev Cell Mol Biol* **2019**, *346*, 97.

[28] R. Di Micco, V. Krizhanovsky, D. Baker, F. D'Adda Di Fagagna, *Nat Rev Mol Cell Biol* **2021**, *22*, 75.

[29] M. Badran, C. Puech, A. Khalyfa, R. Cortese, K. Cataldo, Z. Qiao, D. Gozal, *AMERICAN JOURNAL OF RESPIRATORY AND CRITICAL CARE MEDICINE* **2024**, *209*, 1001.

[30] X. Liu, Y. Gu, S. Kumar, S. Amin, Q. Guo, J. Wang, C. Fang, X. Cao, M. Wan, *Cell Metab* **2023**, *35*, 667.

[31] J. Crouch, M. Shvedova, R. J. R. S. Thanapaul, V. Botchkarev, D. Roh, *Cells* **2022**, *11*.

[32] X. Zhu, Z. Chen, W. Shen, G. Huang, J. M. Sedivy, H. Wang, Z. Ju, *Signal Transduct Target Ther* **2021**, *6*, 245.

[33] K. E. Stephens, C. A. Miaskowski, J. D. Levine, C. R. Pullinger, B. E. Aouizerat, *Biol Res Nurs* **2013**, *15*, 373.

[34] J. Park, K. Lee, K. Kim, S. Yi, *Signal Transduct Target Ther* **2022**, *7*, 217.

[35] A. Meissner, *Nat Biotechnol* **2010**, *28*, 1079.

[36] J. L. Miller, P. A. Grant, *Subcell Biochem* **2013**, *61*, 289.

[37] Y. Fukui, Y. Hirota, S. Aikawa, A. Sakashita, R. Shimizu-Hirota, N. Takeda, C. Ishizawa, R. Iida, T. Kaku, T. Hirata, T. Hiraoka, S. Akaeda, M. Matsuo, Y. Osuga, *Cell Death Dis* **2023**, *14*, 320.

[38] E. Lavarone, C. M. Barbieri, D. Pasini, *Nat Commun* **2019**, *10*, 1679.

[39] F. Cheng, H. Li, J. Liu, F. Yan, Y. Chen, H. Hu, *Genes Immun* **2022**, *23*, 141.

[40] A. Dudakovic, E. T. Camilleri, C. R. Paradise, R. M. Samsonraj, M. Gluscevic, C. A. Paggi, D. L. Begun, F. Khani, O. Pichurin, F. S. Ahmed, R. Elsayed, M. Elsalanty, M. E. Mcgee-Lawrence, M. Karperien, S. M. Riester, R. Thaler, J. J. Westendorf, A. J. van Wijnen, *J Biol Chem* **2018**, *293*, 12894.

[41] C. Li, Y. Chai, L. Wang, B. Gao, H. Chen, P. Gao, F. Zhou, X. Luo, J. L. Crane, B. Yu, X. Cao, M. Wan, *NATURE COMMUNICATIONS* **2017**, *8*, 15.

[42] M. W. Luczak, A. Zhitkovich, *Toxicol Appl Pharmacol* **2017**, *331*, 94.

[43] L. Sun, Y. Ma, H. Niu, Y. Liu, Y. Yuan, C. Liu, *ADVANCED FUNCTIONAL MATERIALS* **2021**, *31*, 1.

[44] J. C. H. Leijten, L. S. Moreira Teixeira, E. B. M. Landman, C. A. van Blitterswijk, M. Karperien, *PLoS One* **2012**, *7*, e49896.

[45] H. Lee, C. Chang, M. Shieh, J. Wang, Y. Chen, T. Young, S. Hung, *Sci Rep* **2013**, *3*, 2683.

[46] E. Noirrit-Esclassan, M. Valera, F. Tremollieres, J. Arnal, F. Lenfant, C. Fontaine, A. Vinel, *INTERNATIONAL JOURNAL OF MOLECULAR SCIENCES* **2021**, *22*, 18.

[47] S. C. Manolagas, *JOURNAL OF BONE AND MINERAL RESEARCH* **2018**, *33*, 371.

[48] J. N. Farr, M. Xu, M. M. Weivoda, D. G. Monroe, D. G. Fraser, J. L. Onken, B. A. Negley, J. G. Sfeir, M. B. Ogrodnik, C. M. Hachfeld, N. K. Lebrasseur, M. T. Drake, R. J. Pignolo, T. Pirtskhalava, T. Tchkonia, M. J. Oursler, J. L. Kirkland, S. Khosla, *Nat Med* **2017**, *23*, 1072.

[49] Y. Cai, Y. Zhang, Y. P. Loh, J. Q. Tng, M. C. Lim, Z. Cao, A. Raju, E. Lieberman Aiden, S. Li, L. Manikandan, V. Tergaonkar, G. Tucker-Kellogg, M. J. Fullwood, *Nat Commun* **2021**, *12*, 719.

[50] T. Shi, D. M. K. van Soest, P. E. Polderman, B. M. T. Burgering, T. B. Dansen, *FREE RADICAL BIOLOGY AND MEDICINE* **2021**, *172*, 298.

[51] X. H. Tang, M. Milyavsky, I. Shats, N. Erez, N. Goldfinger, V. Rotter, *ONCOGENE* **2004**, *23*, 5759.

[52] H. Hong, J. Hosomichi, H. Maeda, K. Lekvijittada, S. Oishi, Y. Ishida, R. Usumi-Fujita, S. Kaneko, J. Suzuki, K. Yoshida, T. Ono, *Eur J Orthod* **2021**, *43*, 94.

[53] E. Esteller, J. C. Villatoro, A. Aguero, R. Lopez, E. Matino, J. Argemi, M. Girabent-Farres, *Int J Pediatr Otorhinolaryngol* **2018**, *108*, 214.

[54] X. Liu, Y. Chai, G. Liu, W. Su, Q. Guo, X. Lv, P. Gao, B. Yu, G. Ferbeyre, X. Cao, M. Wan, *NATURE COMMUNICATIONS* **2021**, *12*, 20.

[55] A. N. Rindone, X. Liu, S. Farhat, A. Perdomo-Pantoja, T. F. Witham, D. L. Coutu, M. Wan, W. L. Grayson, *Nat Commun* **2021**, *12*, 6219.
